# Superb water splitting activity of the electrocatalyst Fe_3_Co(PO_4_)_4_ designed with computation aid

**DOI:** 10.1038/s41467-019-13050-3

**Published:** 2019-11-15

**Authors:** Siraj Sultan, Miran Ha, Dong Yeon Kim, Jitendra N. Tiwari, Chang Woo Myung, Abhishek Meena, Tae Joo Shin, Keun Hwa Chae, Kwang S. Kim

**Affiliations:** 1 Center for Superfunctional Materials, Department of Chemistry, Ulsan National Institute of Science and Technology (UNIST), 50 UNIST-gil, Ulsan, 689-798 Korea; 2 Department of Energy and Chemical Engineering, UNIST, Ulsan, Korea; 3 UNIST Central Research Facilities, UNIST, Ulsan, Korea; 4 Advanced Analysis Center, Korea Institute of Science and Technology, 5 Hwarangno 14-gil, Seongbuk-gu Seoul, 02792 Korea

**Keywords:** Catalyst synthesis, Electrocatalysis, Graphene

## Abstract

For efficient water splitting, it is essential to develop inexpensive and super-efficient electrocatalysts for the oxygen evolution reaction (OER). Herein, we report a phosphate-based electrocatalyst [Fe_3_Co(PO_4_)_4_@reduced-graphene-oxide(rGO)] showing outstanding OER performance (much higher than state-of-the-art Ir/C catalysts), the design of which was aided by first-principles calculations. This electrocatalyst displays low overpotential (237 mV at high current density 100 mA cm^−2^ in 1 M KOH), high turnover frequency (TOF: 0.54 s^−1^), high Faradaic efficiency (98%), and long-term durability. Its remarkable performance is ascribed to the optimal free energy for OER at Fe sites and efficient mass/charge transfer. When a Fe_3_Co(PO_4_)_4_@rGO anodic electrode is integrated with a Pt/C cathodic electrode, the electrolyzer requires only 1.45 V to achieve 10 mA cm^−2^ for whole water splitting in 1 M KOH (1.39 V in 6 M KOH), which is much smaller than commercial Ir-C//Pt-C electrocatalysts. This cost-effective powerful oxygen production material with carbon-supporting substrates offers great promise for water splitting.

## Abstract (web summary)

The sluggish kinetics of the oxygen evolution reaction (OER) is the main obstacle in water splitting which is generally catalyzed by precious metals. Here, authors report a DFT predicted non-precious bimetallic phosphate electrocatalyst that displays high OER activity and stability.

## Introduction

The oxygen evolution reaction (OER) is a fundamental reaction in electrochemical energy conversion process, which is the basis of water splitting, batteries, and photoelectrochemical cells^1–3^. The water splitting is considered as a promising and renewable method for producing hydrogen and oxygen gases^4–7^. However, the efficiency of water splitting in basic electrolyte is largely hindered by sluggish kinetics of the oxidative half-cell OER reaction^2,7^ and so commercial water splitting usually works at a high voltage of 1.8–2.0 V^8^. Recently, noble metal-based compounds such as IrO_2_ and RuO_2_ exhibit good catalytic activities toward OER^9,10^. However, the widespread practical application of these noble metal-based compounds is restricted due to their skyrocketing price and scarcity^5,9^. Therefore, the development of cost-effective catalysts with high electrocatalytic activity and stability for OER is in high demand, which would lead to a cost-effective production of oxygen via water splitting. In this regard, inexpensive earth-abundant transition metal-based OER electrocatalysts would be a good choice not only because of their high abundance and low cost but also due to their high electrocatalytic activity and stability in wide pH ranges^11–13^.

Over the past decade, transition metal-based OER electrocatalysts (for instance, cobalt phosphate, surface-oxidized steels, NiFe-layered double hydroxide/nickel foam, oxy-hydroxides, oxide, perovskites, cobalt phosphate composites, and Co_3_O_4_, etc.) have been explored due to their high potential for water oxidation, high durability under basic condition, and their benign environmental nature^5,7–9,12–14^. Nevertheless, these materials exhibit large overpotential (*η*) for OER^5,7–9^. Therefore, effective designing of state-of-the-art electrocatalyst and clear understanding of OER catalytic mechanism remain challenging tasks.

Herein, we report a phosphate-based electrocatalyst of Fe_3_Co(PO_4_)_4_/reduced-graphene-oxide (rGO) (**1**) for OER, which is predicted to be highly active by density functional theory (DFT). The as-synthesized **1** indeed serves as a highly active electrocatalyst for OER in basic media with overpotential of ~237 mV at 100 mA cm^−2^, and long-term durability (5000 cycles). On the basis of theoretical modeling and experimental observations, the high OER activity of the designed electrocatalyst is ascribed to the PO_4_-induced positive shift of the redox potential. The efficient mass and charge transfer due to defects/dislocations in the PO_4_-induced mix phase and large Brunauer–Emmett–Teller (BET) surface area also help in the OER activity. When it was integrated into asymmetric two-electrode water-splitting cells, the electrolyzer required a potential ~1.45 V in 1 M KOH (or ~1.39 V in 6 M KOH), to drive a current density of 10 mA cm^−2^ for whole water splitting, which is much smaller than that of the integrated commercial Ir-C//Pt-C electrocatalysts (~1.53 V in 1 M KOH).

## Results and discussion

### Theoretical model

**Fig. 1:**
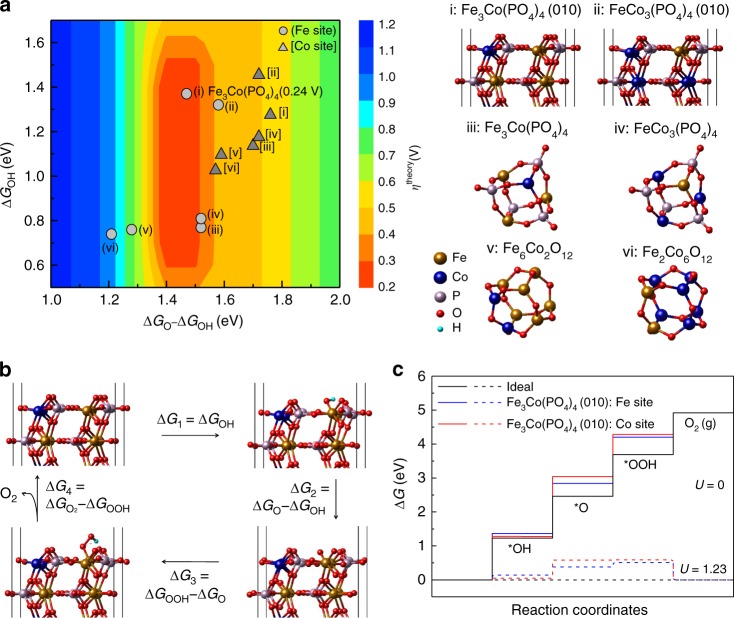
DFT-predicted structures, overpotentials, and free-energy profiles. **a** 2D color-coded map of theoretical overpotential *η*^theory^ as function of free energies Δ*G*_O_ − Δ*G*_OH_ and Δ*G*_OH_ for various compositions (i: Fe_3_Co(PO_4_)_4_ (010), ii: FeCo_3_(PO_4_)_4_ (010), iii: Fe_3_Co(PO_4_)_4_ cluster, iv: FeCo_3_(PO_4_)_4_ cluster, v: Fe_6_Co_2_O_12_ cluster, vi: Fe_2_Co_6_O_12_ cluster) at Fe (gray circle) and Co (dark gray triangle) active sites. **b** Optimized geometries of the *O, *OH, and *OOH intermediates on the Fe sites of Fe_3_Co(PO_4_)_4_ (010) (Supplementary Table 1). **c** Free-energy profiles of OER at zero and equilibrium (1.23 V) potentials for Fe and Co sites of Fe_3_Co(PO_4_)_4_ (010). OER typically undergoes a four-electron step process in alkali media. In an ideal case, the free-energy changes by 1.23 V at each step (black line). The conversion of *OH to *O is the rate-determining step on both Fe site (∆*G*_1_ = 0.24 V) and Co site (∆*G*_2_ = 0.53 V). Source data for **a** and **c** are provided as a Source Data file

Inexpensive Fe and Co hybrids with inorganic species (such as P or O) can show good OER performance^15–17^ in that the covalent strength of Fe/Co-X in a Fe/Co-X-Y linkage is controlled by the inductive effect^16^. Positive shift of the redox potential for transition metal oxide catalysts can lead to high catalytic effect^18^. In consideration of the inductive effect, we have considered various phosphorus oxides P_*x*_O_*y*_ as a better alternative for P/O. Given that O is highly electronegative, substituting O with phosphate would be a good choice for OER. Thus, tuning the catalytic effect would be possible by substituting O with phosphate and further fine-tuning by optimizing the Fe/Co ratio. In many cases, Fe and Co behave similarly with similar ionization potentials *E*_IP_(Fe/Co) = 7.87/7.86 eV but they show a large difference in valence electron configurations [Ar]3d^6^4s^2^/[Ar]3d^7^4s^2^ and electron affinities *E*_EA_(Fe/Co) = 0.15/0.66 eV. Therefore, their cations with varying Fe/Co ratio give different electrochemical properties with different induction effects. This led us to theoretically investigate the OER performance of stable cage structures of Fe_*m*_Co_8-*m*_O_12_ (*m* = 0,2,4,6,8) and Fe_*n*_Co_4-*n*_(PO_4_)_4_ (*n* = 0–4) (Fig. 1a) at varying compositions of Fe and Co (a few varying Fe/Co ratios of 4/0, 3/1, 2/2, 1/3, and 0/4). DFT calculations were performed to understand their electronic structure (Supplementary Figs. 1–4) and *O/*OH free energies (Δ*G*_O_/Δ*G*_OH_) and theoretical overpotentials (*η*^theory^) required for OER (Fig. 1) at various compositions (Supplementary Table 1), where * denotes an active site. We found that the metal substrate of rGO, although significantly beneficial for durability and conductivity (Supplementary Fig. 5), does not give significant effects on H-adsorption energies of Fe_*n*_Co_4-*n*_(PO_4_)_4_ (*n* = 0–4). To study the catalytic effect, here we have focused on the most stable (010) surface of Fe_*n*_Co_4-*n*_(PO_4_)_4_ (Supplementary Table 2) rather than Fe_*n*_Co_4-*n*_(PO_4_)_4_@rGO for the realistic model.

The optimal catalytic activity is found from the Fe/Co ratio of 3 for the Fe_*n*_Co_4-*n*_(PO_4_)_4_ models of clusters and (010) surfaces. For Fe/Co mixed-metal phosphates, the *η*^theory^ at Fe sites is the smallest at *n* = 3, i.e., Fe_3_Co(PO_4_)_4_, whereas the *η*^theory^ of Co sites is not small, remaining almost same for *n* = 0–4 (Supplementary Table 1). In the Fe_*m*_Co_8-*m*_O_12_ (*m* = 0,2,4,6,8) model, the substitution effect of Co for Fe improves the *η*^theory^ at Fe sites but not at Co sites. Thus, in both models, a small amount of Co that substitutes Fe is effective on Fe sites but not so on Co sites, and the optimized Fe/Co ratio at Fe sites in Fe_*n*_Co_4-*n*_(PO_4_)_4_ is 3. The binding energies of intermediate states at Fe sites become weaker if they are surrounded by many Co atoms. As a result, those local Fe sites become less active. The Fe sites of Fe_3_Co(PO_4_)_4_ are predicted to show excellent activity with *η*^theory^ = 0.24 V. The Fe and Co sites are tri-coordinated in clusters and penta-coordinated in (010) surfaces. The binding energies of intermediate states become weaker on the penta-coordinated Fe sites constantly (by ~0.5 eV) than on the tri-coordinated Fe sites. Yet, their free-energy changes between O-adsorption and OH-adsorption (Δ*G*_O_ − ΔG_OH_) behave similarly, except for this constant difference, and so their overpotentials are similar in both models. It seems that the binding energies of all the intermediate states also become weaker at more coordinated sites on surfaces by almost the same magnitude (~0.5 eV) than on clusters.

To sum up, the electronic structure calculations reveal that the phosphate and the Fe/Co ratio (optimal value of 3/1) are important in tuning the redox potential. As compared with Fe_3_CoO_6_ (0001), the phosphate group in Fe_3_Co(PO_4_)_4_ (010) lowers the metal oxide antibonding state and positively shifts the redox potential with the inductive effect (Supplementary Fig. 1). As Co (which is slightly more electronegative than Fe) pulls down the metal oxide antibonding state energy levels and thereby positively shifts the redox potential (Supplementary Fig. 2), tuning the Fe/Co ratio can be exploited for better OER performance.

DFT calculations show that **1** exhibits the optimal Δ*G*_O_ − ΔG_OH_ free energy (Fig. 1a). At the O-adsorbed metal sites of **1**, the bond strength between Fe/Co 3*d* and O 2*p* orbitals becomes weaker than at those of Fe_6_Co_2_O_12_ (Supplementary Fig. 3). Thus, we investigate the energetics of all intermediates (OH, O, OOH adsorption) and evaluate the theoretical overpotentials for OER (*η*^theory^) of catalyst FeCo_3_(PO_4_)_4_ (010) and clusters of Fe_3_Co(PO_4_)_4_, FeCo_3_(PO_4_)_4_, Fe_6_Co_2_O_12_, and Fe_2_Co_6_O_12_ (Fig. 1). Iron-cobalt oxide clusters are also studied to understand their ratio effect on catalytic activity. We calculate *η*^theory^ for the FeCo_3_(PO_4_)_4_ cluster and FeCo_3_(PO_4_)_4_ (010) surface models, and other cases with different Fe/Co ratios. The *η*^theory^ at Co site of FeCo_3_(PO_4_)_4_ (010) is 0.49 V, which is larger than the *η*^theory^ by 0.24 V at Fe site of Fe_3_Co(PO_4_)_4_. The Fe site in the Fe_3_Co(PO_4_)_4_ (010) has a lower Δ*G*_2_ value (1.47 V) than that of the Co site (1.76 V). The improvement in *η*^theory^ at the Fe site in **1** over other considered composites is attributed to optimal Δ*G*_O_ − Δ*G*_OH_ by weakend *O and *OH binding at the Fe site, whereas the same change of *O and *OH binding at Co sites affects the OER reaction only moderately (Fig. 1b,c, Supplementary Fig. 4, and Supplementary Table 1). Overall, our computation shows that **1** reduces the energy barriers for every step, thereby lowering the free energies of each elementary reaction step (Fig. 1b, c and Supplementary Table 1).

### Synthesis and characterization

**Fig. 2:**
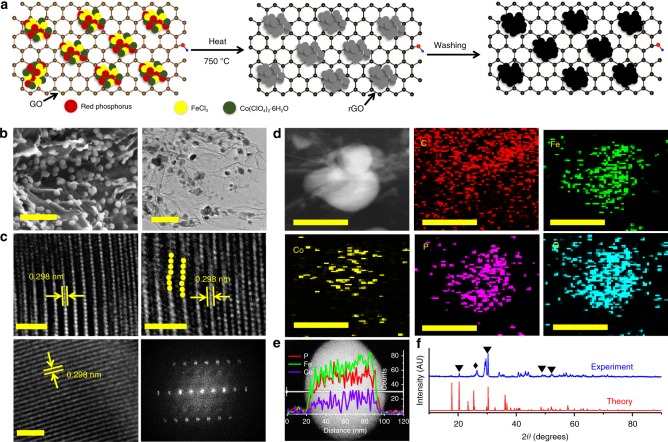
Preparation route, structural, and compositional characterizations of Fe_3_Co(PO_4_)_4_@rGO (**1**). **a** Synthesis procedure: first, mix the sample of graphene oxide, red phosphorus, FeCl_3_, and Co(ClO_4_)_2_·6H_2_O, and then heat. Second, leach with acid, wash with DI water, and dry in the oven. **b** Scanning (left) and transmission (right) electron micrographs. Scale bars, 200 nm. **c** Representative high-resolution transmission electron microscopy (HRTEM) images for different positions of single-particle (yellow dots denote atomic dislocations). Fast Fourier transform (FFT) image is on right-bottom panel. Scale bars, 2 nm. **d** High-angle annular-dark field scanning transmission electron microscope (HAADF–STEM) image and their corresponding individual element maps of C, Fe, Co, P, and O in a part of **1**. **e** HAADF–STEM image with the overlapping image showing the corresponding EDS line-scan profiles. Scale bars, 50 nm. **f** X-ray diffraction pattern, which confirms the formation of Fe_3_Co(PO_4_)_4_ in **1**. The peak marked by black diamond denotes the rGO peak. Additional small unmatched peaks may be resulted from the byproduct adduct of Fe_4.1_Co_2.9_(PO_4_)_6_ and mono-metallic iron or cobalt phosphate (Supplementary Fig. 6). Source data for **e** and **f** are provided as a Source Data file

In light of above findings, we synthesized three catalysts of [**1:** (Fe_3_Co(PO_4_)_4_@rGO), **2:** (Fe_1-1.33_Co(PO_4_)_2_@rGO) (or FeCo(PO_4_)_2_@rGO), and **3:** (Fe_1.5-2_Co(PO_4_)_3_@rGO) (or Fe_2_Co(PO_4_)_3_@rGO)] with a one‐pot temperature‐programmed carbonization process (Fig. 2a and Methods). As **1** shows the best activity, we will focus our discussion on **1**, unless otherwise specified.

The scanning electron microscopy (SEM) and low-resolution transmission electron microscopy (TEM) images of **1** show that Fe_3_Co(PO_4_)_4_ nanoparticles (NPs) are uniformly distributed on the rGO surface with diameters 50–70 nm (Fig. 2b). The high-resolution TEM (HRTEM) and fast Fourier transform (FT) images reveal that the Fe_3_Co(PO_4_)_4_ NPs are crystalline (Fig. 2c). The HRTEM images show *d*-spacing values of 2.98 Å, corresponding to the {020} planes for the Fe_3_Co(PO_4_)_4_ crystal. Plane defects and atomic dislocations (yellow dots in Fig. 2c) enhance the specific area and the electrocatalytic site for further boosting the OER activity. In addition, high-angle annular-dark-field scanning TEM (HAADF–STEM) energy-dispersive spectroscopy mapping was used to examine the elements distribution in Fe_3_Co(PO_4_)_4_@rGO (Fig. 2d). Composition line-scan profiles across an NP of **1** shows that Fe/Co/P elements are distributed throughout the NP (Fig. 2e). The X-ray diffraction (XRD) patterns of **1** show the peaks at 20.3°, 30.3°, 48.52°, and 52.1° (marked as “▼”) corresponding to {101}, {020}, {220}, and {222̅} crystal planes, respectively, for a monoclinic Fe_3_Co(PO_4_)_4_ crystal (space group: Pm) (Fig. 2f and Supplementary Fig. 6). In addition, the {020} peak has the maximum intensity, indicating that the c-axis [010] is the growth direction of Fe_3_Co(PO_4_)_4_ crystal. Both theoretical and experimental patterns are quite similar; however, few peaks slightly blue-shifted in experiment, indicating the volume expansion with increased lattice spacing (due to phosphate intercalation) and crystal defects/dislocations (due to strain effect during the metal cations formation). These observations are in good agreement with the HRTEM results as discussed earlier. The expanded curves of Fe_3_Co(PO_4_)_4_@rGO show a broad band at 26.3° (marked as “♦”) corresponding to the (002) plane of rGO, indicating the successful reduction of GO and the formation of graphitic structures with interlayer spacing of 0.34 nm^19–21^. A few small unmatched diffraction peaks, such as 35‒39°, 41‒42°, 55‒60°, and 63‒80°, are not clear to us, because there can be so many possible structures in small quantities, but may be resulted from a byproduct adduct Fe_4.1_Co_2.9_(PO_4_)_6_, mono-metallic iron, or cobalt phosphate during the one-pot temperature-programmed carbonization process (Supplementary Fig. 6). However, it is clear that they are not a significant issue, because all experimental results are well explained by our theoretical models.

**Fig. 3:**
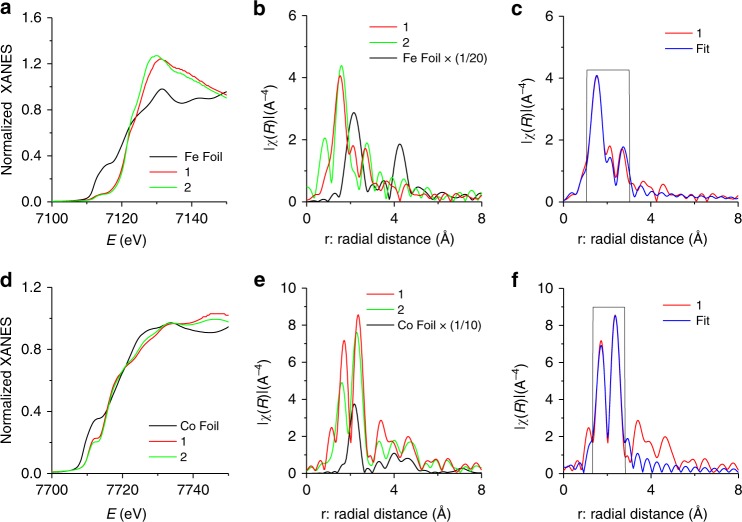
X-ray absorption spectra. **a**–**c** Fe K-edge. **d**–**f** Co K-edge. **a**, **d** XANES spectra for Fe K- and Co-K-edges. **b**, **e** Fourier transform (FT) of the EXAFS spectra in real space at Fe K- and Co K-edges. **c**, **f** FT–EXAFS spectra in r-space and the corresponding least-squares fit (black rectangular line) for first and second shells. **1**: Fe_3_Co(PO_4_)_4_@rGO; **2**: FeCo(PO_4_)_2_@rGO. Source data for **a**–**f** are provided as a Source Data file

We conducted FT X-ray absorption fine structure (FT–EXAFS) analysis, which provided the chemical state and coordination environments^22–25^. The energies of both XANES Fe K-edges and Co K-edges of **1** and FeCo(PO_4_)_2_@rGO (**2**) are positively shifted compared with those of Fe/Co foils (Fig. 3a, d), suggesting the oxidized states of Fe/Co. The FT–EXAFS data of Fe and Co in **1** and **2** exhibit similar peak patterns, but the peak intensities and positions are slightly changed due to their different compositions. Meanwhile, Fe and Co foils show different peak positions for Fe–Fe and Co–Co bonds (Fig. 3b, e). We analyzed the EXAFS curve of **1** using least-square fit for first and second shells. Figure 3c (and Supplementary Fig. 7a) show that the major peaks at 2.0 Å (coordination number (CN): 4.1) and 2.2 Å (CN: 1.9) reflect Fe = O and Fe–O bond distances, while the minor peak reflects the overlapped peaks at Fe^…^P distances (via O) of 2.9/3.1/3.3 Å (CN: 1.6/1/2.4, respectively) (Supplementary Table 3). Similarly, Fig. 3f and Supplementary Fig. 7b show peaks at 2.0 Å (CN: 2) and 2.4 Å (CN: 4) for Co = O and Co–O bond distances, while a major peak at 2.8 Å (CN: 5) for the Co^…^P distance via O (Supplementary Table 4).

We calculated theoretical Fe and Co K-edge EXAFS of Fe_3_Co(PO_4_)_4_ using the FDMX package^26,27^. These theoretical FT–EXAFS spectra of the Co and Fe atoms are similar to the experimental FT–EXAFS spectra of **1** in *r*-space (Supplementary Fig. 8 and Fig. 3c, f). It seems that the difference between Fe and Co K-edge EXAFS of **1** arises from different Fe-P distances at each Fe sites in the crystal (Supplementary Tables 3 and 4). To confirm this, we calculated EXAFS of pure Fe_4_(PO_4_)_4_ and Co_4_(PO_4_)_4_, where the Co K-edge FT–EXAFS spectra of Co_4_(PO_4_)_4_ are similar to the Fe K-edge FT–EXAFS in *r*-space. This indicates that Co atoms well replace Fe sites in the Fe_3_Co(PO_4_)_4_ lattice.

The core level P-2p X-ray photoelectron spectroscopy (XPS) shows two major peaks at binding energies of 133.2 ± 0.1 and 134.1 ± 0.1 eV, corresponding to the 2*p*_3/2_ and 2*p*_1/2_ core levels of central phosphorus atoms in phosphate species^28^, respectively, which is characteristic of the tetrahedral (PO_4_) group^29^ (Supplementary Fig. 9a). Furthermore, the O-1*s* XPS spectra show two peaks at 531.4 and 532.5 eV, assigned to phosphate species^30^ (Supplementary Fig. 9b). The atomic ratio of Fe/Co are 3/1 or 3.1/1, as measured by inductively coupled plasma atomic-emission spectroscopy (ICP-AES) (Supplementary Table 5). In addition, XPS analysis provides the atomic percentage near the sample surface. As compared with ICP-AES bulk sample analysis, the XPS surface analysis increases the Fe(PO_4_) content, whereas the atomic content of P (or PO_4_) is almost the sum of Co and Fe atomic contents, indicating the charges of Co and Fe are +3 (Supplementary Table 6). The content of O is slightly larger than four times of the content of P due to environmental oxygen. An extra content of Fe(PO_4_), as noted from XPS over ICP-AES, could be present on the surface more than in bulk. However, the XPS data are not so reliable for accurate composition analysis as compared with ICP-AES. The Fe/Co metals composition ratio from ICP-AES is more reliable. Overall, the elemental composition turns out to be **1:** Fe_3_Co(PO_4_)_4_@rGO (Fe/Co = 3), **2**: FeCo(PO_4_)_2_@rGO (Fe_4_Co_3_(PO_4_)_7_@rGO) (Fe/Co = 1–1.33), and **3**: Fe_2_Co(PO_4_)_3_@rGO (or Fe_3_Co_2_(PO_4_)_5_@rGO) (Fe/Co = 1.5–2). To facilitate our discussion, **2** and **3** will be simply denoted as **2**: FeCo(PO_4_)_2_@rGO and **3**: Fe_2_Co(PO_4_)_3_@rGO in consideration with the experimental component analysis.

### Electrochemical performance

**Fig. 4:**
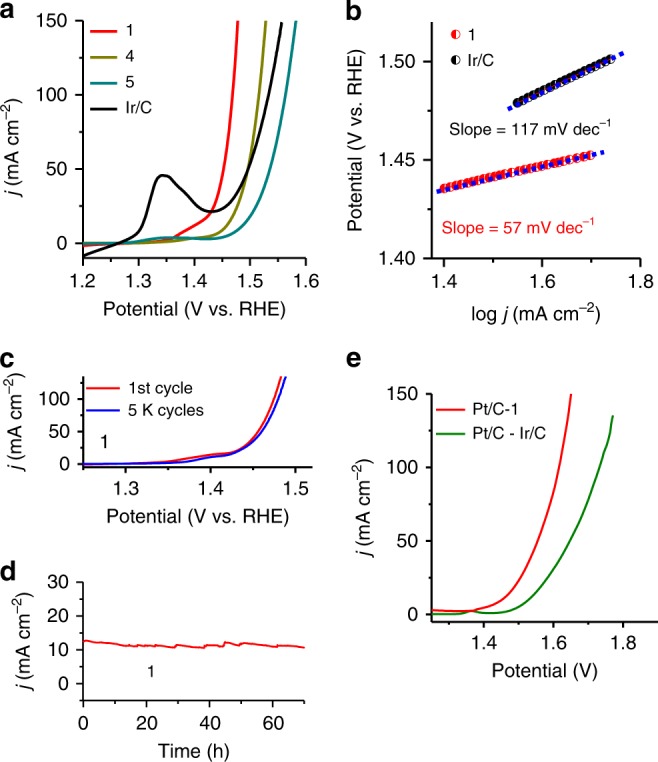
Electrochemical performance of electrocatalysts toward OER and whole-cell water splitting. **a** Linear sweep voltammograms (LSVs) curves measured in 1 M KOH at a scan rate of 5 mV s^−1^. **b** Tafel slope. **c** LSVs recorded at a scan rate of 5 mV s^−1^ during the OER before and after continuous polarization of the electrode in 1 M KOH for 5000 cycles. **d** Chronoamperometry curves recorded in 1 M KOH for 70 h. **e** Current density of Pt/C-**1** vs. Pt/C-Ir/C for overall water splitting in 1 M KOH. **1:** Fe_3_Co(PO_4_)_4_@rGO, **4:** Fe_2_P_2_O_7_)@rGO, **5:** (CoFe_2_O_4_)(Fe_2_O_3_)@rGO. Source data for **a** and **c**–**e** are provided as a Source Data file

We performed electrochemical measurements to check the catalytic activities of catalysts **1–9** and commercial 20 wt% Ir/C for OER in 1 M KOH electrolyte **(**Fig. 4a and Supplementary Figs. 10–12). The OER activity of these electrocatalysts are influenced by the amount of Fe or Co in the presence of phosphate and the best activity is achieved for **1**. Compound **1** exhibits very small overpotential of ~237 mV to afford a current density of 100 mA cm^−2^, lower than catalysts 2–9 and **Ir/C** (Fig. 4a and Supplementary Figs. 10–12), and other catalysts with a carbon-supporting substrate including the benchmark Ir/C catalyst (303 mV). This activity is found to be among the best OER-active catalysts (Supplementary Table 8) among which some catalysts of pure metals without the carbon-supporting substrate^31–33^ do not show high stability (<10 h at 10 mA cm^−2^), except for the special case of core-shell FeNiCu, which shows a promising result with high stability at low overpotential^34^. Nevertheless, in most cases the industrial application still requires carbon substrates.

The TOF^35,36^ is calculated to be 0.54 s^−1^ at an overpotential of 237 mV, indicating a highly active catalyst, 7.6 times that of Ir/C 0.071 s^−1^ (details in Methods), which further confirms the outstanding OER performance of **1**. The Tafel slope is 57 mV per decade (Fig. 4b), smaller than that of Ir/C (117 mV per decade). The small overpotential at Fe sites of **1** is the key factor for the superior OER activity of **1**. For achieving this optimal overpotential, other environmental factors have also been utilized as follows. The large BET surface area (238 m^2^/g) (Supplementary Fig. 13) with an average pore size 4.3 nm promotes the contact between **1** and an electrolyte, thereby helping in the optimal OER activity. The very low charge-transfer resistance (0.29 Ω on a nickel foam (NF) substrate, 7.5 Ω on glassy carbon electrode (GCE) substrate) promotes electron transport, thereby leading to faster kinetics (Supplementary Fig. 14a, b). The electrochemical double-layer capacitance (*C*_*dl*_ = 0.0162F), which is directly correlated to the catalyst’s active surface area, is very large, 8.1-fold the *C*_*dl*_ of Ir/C (0.002F) (Supplementary Fig. 15). The defects/dislocations (strain effect)^37–39^ of Fe and Co atoms in crystal help in improving the performance of active sites.

After initial and 5000 cyclic voltammogram (CV) cycles, accelerated degradation test of **1** indicates the excellent durability as demonstrated by the near overlay of OER curves (Fig. 4c). No significant changes in TEM, EDX (energy-dispersive X-ray spectroscopy), HRTEM, hard/soft XAS, XPS, and Raman spectra (except for minor increase in FeO_*x*_/FeOOH and CoO_*x*_/CoOOH^40–42^, which would not be so active such as Fe sites of **1**, Supplementary Fig. 16) were observed before and after the test, indicating that **1** is quite durable (Supplementary Figs. 17–23). The stability of this catalyst was further assessed by chronoamperometry (CA). Compound **1** exhibited outstanding stability with no changes in current density of ~10–11 mA cm^−2^ (70 h on NF and 55 h on GCE) and ~210 mA cm^−2^ (for 45 h on NF) ((Fig. 4d and Supplementary Fig. 24). The high stability and durability of nanocrystal in **1** is ascribed to the graphene support (which is very stable in alkaline and acid media) and the strong coordination between Fe/Co and PO_4_. Initially and 54 h after the stability test, the Faradaic efficiency (after 1 h test) is ~98 and ~96% in alkaline electrolyte, respectively (Methods).

To evaluate the real application, an overall water-splitting cell was fabricated in which 1 M/6 M KOH was used as electrolyte and **1** served as the anode with 20 wt% Pt/C catalyst as the cathode. It required ~1.45 V in 1 M KOH (1.39 V in 6 M KOH) to facilitate overall water splitting at a current density of 10 mA cm^−2^ (Fig. 4e, Supplementary Fig. 25, and Supplementary Video 1), which is the lowest voltage, much lower than that of the benchmark combination (1.53 V; commercial Pt/C and Ir/C catalysts). It required nominal voltage of 1.4 V to drive overall water splitting (Supplementary Fig. 26).

We report the SEM, TEM, HRTEM, and EDX images of catalysts **2**: FeCo(PO_4_)_2_@rGO] and **3**: Fe_2_Co(PO_4_)_3_@rGO] in Supplementary Figs. 27 and 28, and Supplementary Note 2. The structural and compositional characterizations of **2**, **3**, and **5–9** (XRD and XPS) are also provided in Supplementary Figs. 29–37.

Finally, for further optimization of catalysts, one may explore some other P_*x*_O_*y*_ instead of PO_4_. Further elucidation of the clear origin for the optimal Fe/Co ratio would help in future design of other metal cations electrocatalysts.

In summary, we synthesized **1** and systematically evaluated their OER catalytic activities in alkaline conditions. This hybrid exhibited excellent OER catalytic activities (very low overpotential of 237 mV at high current density 100 mA cm^−2^) and outstanding stabilities, which is superior to that of the benchmark Ir/C. The remarkable performance is attributed to the PO_4_ groups, which reduce free-energy barrier for OER at Fe and Co sites, and increase mass/charge transfer arising from defects/dislocation in the PO_4_-induced mix phase. As a full water splitting, we fabricated an electrolyzer with this hybrid catalyst in alkaline condition, which afforded a current density of 10 mA cm^−2^ with ~1.45 V in 1 M KOH (~1.39 V in 6 M KOH). This work demonstrates the potential of large-scale structure-engineering towards low-cost, earth-abundant, and high-performance water splitting for energy applications.

## Methods

### Chemicals

Cobalt(II) perchlorate hexahydrate, iron (III) chloride (reagent grade, 97%), red phosphorus (reagent grade, 99.99%), 5 wt% of Nafion, and perchloric acid were purchased from Sigma-Aldrich. Benchmark 20 wt% Ir/C (commercial) and 20 wt% Pt/C (commercial) catalysts were purchased from Johnson Matthey and Premetek, Co. All the chemicals were of analytical grade and were used as received without further purification.

### Synthetic procedure

In synthesis, 200 mg of graphene oxide (GO) and 70 mg of red phosphorus with different weight ratios of iron chloride (FeCl_3_) to cobalt perchlorate hexahydrate Co(ClO_4_)_2_.6H_2_O were used as starting materials. During the synthesis process, 200 mg of GO was dispersed in 100 mL double distilled water by sonication. The precursors’ weight ratios were chosen as 0.70 g FeCl_3_, 0.35 g Co(ClO_4_)_2_.6H_2_O, and 0.07 g red phosphorus for **1** [1: Fe_3_Co (PO_4_)_4_@rGO], 0.6 g FeCl_3_, 0.45 g Co(ClO_4_)_2_.6H_2_O, and 0.07 g red phosphorus for **2** [**2**: Fe_1-1.33_Co(PO_4_)_2_@rGO or FeCo(PO_4_)_2_@rGO], and 0.65 g FeCl_3_, 0.4 g Co(ClO_4_)_2_.6H_2_O, and 0.07 g red phosphorus for **3** [**3**: Fe_1.5-2_Co(PO_4_)_4_@rGO or Fe_2_Co(PO_4_)_4_@rGO]. The resulting solutions were sonicated for 10 h to obtain fine homogeneous mixture. The mixture was then dried and thoroughly ground. The ground powder was annealed at 750 °C for 3 h with a temperature ramping rate of 3 °C min^−1^ in nitrogen atmosphere. To remove the inactive species, the obtained product was leached with 0.1 M HClO_4_ for 10 h, then filtered and washed with ethanol and water, and finally dried in vacuum at 60 °C. For the synthesis of **4** and **5** (**4**: Fe_2_P_2_O_7_@rGO, **5**: (CoFe_2_O_4_)(Fe_2_O_3_)@rGO), the same method was employed using 0.70 g FeCl_3_ and 0.07 mg red phosphorus for **4**, and 0.70 g FeCl_3_ and 0.35 g Co(ClO_4_)_2_.6H_2_O for **5**. To study the effect of GO and red phosphorous on the OER performance, we have synthesized catalysts with different amount of GO and red phosphorous such as **6** [**6**: 0.7 g FeCl_3_, 0.35 g Co(ClO_4_)_2_.6H_2_O, 100 mg GO, and 0.07 g red phosphorus], **7** [**7**: 0.7 g FeCl_3_, 0.35 g Co(ClO_4_)_2_.6H_2_O, 300 mg GO, and 0.07 g red phosphorus], **8** [**8**: 0.7 g FeCl_3_, 0.35 g Co(ClO_4_)_2_.6H_2_O, 200 mg GO, and 0.035 g red phosphorus], and **9** [**9**: 0.7 g FeCl_3_, 0.35 g Co(ClO_4_)_2_.6H_2_O, 200 mg GO, and 0.140 g red phosphorus]. The C and P contents obtained from XPS analysis in catalysts **6–9** are shown in Supplementary Table 7.

### Characterization techniques

The cold field-emission SEM images were taken using Hitachi High-Technologies S-4800 microscope. TEM and HRTEM images and HAADF–STEM images were taken on JEM-2100F with 200 kV acceleration voltage. XPS data were carried out on K-alpha (Thermo Fisher, UK) system. The surface area, pore size distribution, and pore volume with N_2_ adsorption/desorption isotherms from BET technique were measured on BELSORP-miniII (BEL Japan, Inc.) system. The atomic and weight percent of metals and non-metals in each synthesized catalyst were obtained with ICP-AES (700-ES, Varian) and XPS. EXAFS analysis was performed on ionization detectors at the Pohang Accelerator Laboratory (PAL). The X-ray absorption spectra for the Fe K-edge and Co K-edge were acquired at room temperature using beamlines 6D and 10C (PAL), where their X-ray energies from EXAFS analysis were calibrated with Fe-foil and Co-foil, respectively. Background subtraction, normalization, and FT were done by standard procedure with ATHENA program^43^. The extracted EXAFS signal, *χ*(*r*) and *k*^3^*χ*(*k*) were analyzed for all three metals. The Artemis program was used for EXAFS fitting. Soft XAS measurements were performed at the soft X-ray 10D XAS KIST beamline operating at 3.0 GeV with a maximum storage current of 360 mA. XAS spectra for Fe and Co L_3,2_-edge were collected in the total electron yield mode at room temperature in vacuum of ~1.5 × 10^−8^ Torr. All the spectra were background subtracted and normalized with respect to the incident photon flux measured by inserting a gold (Au) mesh in the path of the X-ray beam.

### Electrochemical characterization

All electrochemical measurements were performed on VSP instrument (BioLogic Science Instruments, Inc.) with three-electrode setup using graphite rod and Hg/HgO as a counter and reference electrodes, respectively. The working electrodes in our experiment were GCE (0.0706 cm^2^) and NF with a geometric area of 3 cm^2^. For working electrode preparation, 1 mg cm^−2^ loading amount of the catalyst was achieved through a drop casting method. To ensure the H_2_O/O_2_ equilibrium during the electrochemical measurement for OER, the electrolyte (1 M KOH) was saturated with a continuous flow of oxygen for 20 min. For stabilizing the working electrodes, the CVs were first conducted at a sweep rate of 100 mV s^−1^ in the potential range of 1.1–1.7 V vs. reversible hydrogen electrode (RHE). The linear sweep voltammograms test were performed at a scan rate of 5 mV s^−1^ with 100% iR compensation, which was automatically derived from the electrochemical workstation. The cycling durability test was carried out at a scan rate of 100 mV s^−1^ for 5000 CV cycles in the potential range of 1.2–1.6 V vs. RHE. CA responses were performed on both GC (for 55 h) and NF (for 70 h) substrates at potentials on which the current densities reached to the range of 10 mA cm^−2^. To evaluate the durability of **1** for high current density generation, the CA response were performed on NF substrate at a current density of ~210 mA cm^−2^ for 45 h. All the CA response were performed without iR compensation. Polarization curves obtained before and after the durability CV cycles were collected for comparison. The current density (mA cm^−2^) was normalized to the electrode geometrical area and the potentials recorded vs. Hg/HgO (1 M NaOH) were converted to RHE according to the reference electrode calibration value (Supplementary Note 1 and Supplementary Fig. 38). For double-layer capacitance (*C*_*dl*_) measurements, the potential in a non-Faradic region (1.059–1.174 V vs. RHE) were cycled at a different applied scan rate of 10, 20, 30, 40, and 50 mV s^−1^. The current from CV curves was plotted vs. applied scan rate at a potential of 1.125 V and the slope obtained from the straight line of current and scan rate were assigned to *C*_*dl*_. The electrochemical impedance spectroscopy (EIS) was performed at an overpotential of 0.30 V vs. RHE in a frequency range of 100 kHz to 0.01 Hz with a modulation amplitude of 10 mV in 1 M KOH solution. The full water splitting was measured in a two-electrode setup with 1 as an OER electrode and Pt/C as a HER catalyst in 1 M KOH (the catalyst loading was 1.5 mg cm^−2^) and 6 M KOH (the catalyst loading was 5 mg cm^−2^) solutions.

The Tafel plots and slopes were calculated according to the Eq. (1):

1 $${\mathrm{\eta }} = {{b}}\,{\mathrm{log}}\,{{j}} + {{C}}$$

where *η*, *j*, *b*, and *C* represent overpotential, current density, Tafel slope, and intercept, respectively.

The overpotential was calculated according to the Eq. (2):

2 $${\mathrm{\eta }} = {{E}}\left( {{\mathrm{vs}}.{\mathrm{RHE}}} \right) - 1.23$$

*TOF of catalyst*: The number of oxygen turnovers was calculated from the current density using the Eq. (3)^35^:

3 $${\mathrm{TOF}} = \frac{{{\mathrm{Total}}\,{\mathrm{number}}\,{\mathrm{of}}\,{\mathrm{O}}_2\,{\mathrm{turnovers}}/{\mathrm{geometric}}\,{\mathrm{area}}({\mathrm{cm}}^2)}}{{{\mathrm{Number}}\,{\mathrm{of}}\,{\mathrm{active}}\,{\mathrm{sites}}/{\mathrm{geometric}}\,{\mathrm{area}}({\mathrm{cm}}^2)}}$$

$$\begin{array}{*{20}{l}} {{\mathrm{Number}}\,{\mathrm{of}}\,{\mathrm{O}}_2\,{\mathrm{turnovers}}} \hfill & = \hfill & {\left( {{\mathrm{j}}\frac{{{\mathrm{mA}}}}{{{\mathrm{cm}}^2}}} \right)\left( {\frac{{1\frac{{\mathrm{C}}}{{\mathrm{s}}}}}{{1000\,{\mathrm{mA}}}}} \right)\left( {\frac{{1\,{\mathrm{mol}}\,{\mathrm{e}}^ - }}{{96485\,{\mathrm{C}}}}} \right)\left( {\frac{{1\,{\mathrm{mol}}\,{\mathrm{O}}_2}}{{4\,{\mathrm{mol}}\,{\mathrm{e}}^ - }}} \right)\left( {\frac{{6.022 \times 10^{23}{\mathrm{mol}}\,{\mathrm{O}}_2}}{{1\,{\mathrm{mol}}\,{\mathrm{O}}_2}}} \right)} \hfill \\ {} \hfill & = \hfill & {1.56 \times 10^{15}\left( {\frac{{{\mathrm{O}}_2/{\mathrm{s}}}}{{{\mathrm{cm}}^2}}} \right){\mathrm{per}}\left( {\frac{{{\mathrm{mA}}}}{{{\mathrm{cm}}^2}}} \right)} \hfill \end{array}$$

The number of Fe and Co ions in **1** was obtained from the ICP analysis ~28.9 wt%. Consequently, the density of active sites based on bulk Fe and Co is:

$$\left( {\frac{{28.9\,{\mathrm{mg}}}}{{100\,{\mathrm{mg}}}}} \right) \times \left( {\frac{{1\,{\mathrm{mg}}}}{{{\mathrm{cm}}^2}}} \right) \times \left( {\frac{{1\,{\mathrm{mmole}}}}{{606.35{\mathrm{mg}}}}} \right) \times 6.022 \times 10^{20}\left( {\frac{{{\mathrm{sites}}}}{{{\mathrm{mmole}}}}} \right) = 2.87 \times 10^{17}{\mathrm{sites}}/{\mathrm{cm}}^2$$

The TOF of the catalyst at an overpotential of 237 mV (current density 100 mA cm^−2^) was calculated as:

$$\frac{{1.56 \times 10^{15}\left( {\frac{{{\mathrm{O}}_2/{\mathrm{s}}}}{{{\mathrm{cm}}^2}}} \right){\mathrm{per}}\left( {\frac{{{\mathrm{mA}}}}{{{\mathrm{cm}}^2}}} \right) \times 100\left( {\frac{{{\mathrm{mA}}}}{{{\mathrm{cm}}^2}}} \right)}}{{2.87 \times 10^{17}{\mathrm{sites}}/{\mathrm{cm}}^2}} = 0.54/{\mathrm{site}}\,{\mathrm{s}}^{ - 1}$$

*Faradaic efficiency*: Faradaic efficiency was calculated using the Eq. (4):

4 $${\mathrm{Faradaic}}\,{\mathrm{efficiency}} = \frac{{{\mathrm{experimental}}\,{\mathrm{\mu mol}}\,{\mathrm{of}}\,{\mathrm{O}}_2\,{\mathrm{gas}}}}{{{\mathrm{theoretical}}\,{\mathrm{\mu mol}}\,{\mathrm{of}}\,{\mathrm{O}}_2\,{\mathrm{gas}}}} \times 100$$

The theoretical amount of O_2_ gas was calculated from Faraday’s law Eq. (5):

5 $${{n}} = \frac{{{{I}} \times {{t}}}}{{{{z}} \times {{F}}}}$$

where *n* is the number of mol, *I* is the current in ampere, *t* is the time in seconds, *z* is the transfer of electrons (for O_2_ *z* = 4), and *F* is the Faraday constant (96,485 C mol^−1^).

The theoretical amount of O_2_ gas before stability = 19.52 µmol.

The theoretical amount of O_2_ gas after stability = 19.00 µmol.

The experimental amount of O_2_ gas was evaluated from the water displacement method using the following protocol:

In our calculations, the pressure is converted into units of an atmosphere by Dalton’s law of partial pressure Eq. (6):

6 $${{P}}_{{\mathrm{Total}}} = {{P}}_{{\mathrm{oxygen}}} + {{P}}_{{\mathrm{water}}}$$

At ambient conditions, the vapor pressure of water is 21.1 mm Hg (this valve was chosen from vapor pressure table).

$$\begin{array}{*{20}{l}} {762\,{\mathrm{mmHg}}} \hfill & = \hfill & {{{P}}_{{\mathrm{oxygen}}} + 21.1\,{\mathrm{mmHg}}} \hfill \\ {{{P}}_{{\mathrm{oxygen}}}} \hfill & = \hfill & {762\,{\mathrm{mmHg}} - 21.1\,{\mathrm{mmHg}}} \hfill \\ {{{P}}_{{\mathrm{oxygen}}}} \hfill & = \hfill & {740.9\,\mathrm {mmHg}} \hfill \end{array}$$

The pressure in our calculations with respect to one atmosphere was:

$$\left( {740.9\,{\mathrm{mmHg}}} \right)(1\,{\mathrm{atm}}/760\,{\mathrm{mmHg}}) = 0.975\,{\mathrm{atm}}$$

Finally, the number of mol oxygen gas produced in water displacement is calculated by the Eq. (7):

7 $${{PV}} = {{nRT}}$$

*V* is the volume of produced gas in liters, *T* is the temperature in kelvin, and *R* is the ideal gas constant (0.0821 L atm/mol K).

The number of moles oxygen gas produced in water displacement before the stability:

$$(0.975\,{\mathrm{atm}})(0.00048{\mathrm{L}}) =	 \, {{n}}\,\left( {0.0821\,{\mathrm{L}}\,{\mathrm{atm}}/{\mathrm{mol}}\,{\mathrm{K}}} \right)\left( {298\,{\mathrm{K}}} \right) \\ {{n}} =	 \, \frac{{\left( {0.975\,{\mathrm{atm}}} \right) \times (0.00048\,{\mathrm{L}})}}{{(0.0821\,{\mathrm{L}}\,{\mathrm{atm}}/{\mathrm{mole}}\,{\mathrm{K}}) \times (298{\mathrm{K}})}} \\ {{n}} =	 \, 19.1\,{\mathrm{\mu}}\,{\mathrm{mol}}$$

The number of mol oxygen gas produced in water displacement after the stability:

$$(0.975\,{\mathrm{atm}})(0.00046{\mathrm{L}}) =	 \,{\mathrm{n}}\left( {0.0821{\mathrm{Latm}}/{\mathrm{mole}}\,{\mathrm{K}}} \right)\left( {298{\mathrm{K}}} \right) \\ {\mathrm{n}} =	 \,\frac{{\left( {0.975\,{\mathrm{atm}}} \right) \times (0.00046{\mathrm{L}})}}{{(0.0821\,{\mathrm{L}}\,{\mathrm{atm}}/{\mathrm{mole}}\,{\mathrm{K}}) \times (298{\mathrm{K}})}} \\ {\mathrm{n}} =	 \,18.3\,{\mathrm{\mu }}\,{\mathrm{mol}}$$

Faradaic efficiency before stability.

$${\mathrm{Faradaic}}\,{\mathrm{efficiency}} = \frac{{19.10}}{{19.52}} \ast 100$$

Faradaic efficiency after stability.

$${\mathrm{Faradaic}}\,{\mathrm{efficiency}} = \frac{{18.30}}{{19.00}} \ast 100$$

The Faradaic efficiency before stability was 98%, whereas it was 96% after the 54 h of chronoamperometric stability test.

*Computational method*: Spin-polarized calculations were performed to obtain ground DFT energies of Fe_*m*_Co_8-*m*_O_12_ (*m* = 0,2,4,6,8) clusters, Fe_*n*_Co_4-*n*_(PO_4_)_4_ (*n* = 0–4) clusters, and Fe_*n*_Co_4-*n*_(PO_4_)_4_ (010) surfaces using Vienna ab initio simulation package^44^ with projected augmented wave functional^45,46^. The exchange-correlation potential was corrected by Perdew, Burke, and Ernzerhof (PBE)^47^ for generalized gradient approximation with Tkatchenko–Scheffler^48^ (TS) dispersion correction (PBE + TS). For structure optimization, a 2 × 2 *k*-grid (1 × 1 *k*-grid for clusters) was used to sample the first Brillouin zone and the cut-off energy was set to 500 eV. Self-consistent field calculations were used to optimize electronic and ionic steps until the energy difference between two successive steps becomes 0.01 meV and the force constant on each ion reaches to 0.02 eV Å^−1^. Calculations were optimized with spin-polarized method, resulting in the high-spin ferromagnetic configuration for Fe_*n*_Co_4-*n*_(PO_4_)_4_ (010) surfaces (Supplementary Table 9) and the most stable magnetic configuration was also considered for clusters models. These ferromagnetic configurations are in agreement with the experimentally verified ferromagnetic magnetic materials of catalysts **1–3**.

The Gibbs free-energy changes (Δ*G*) for OER mechanism in alkaline environment are as follows (equations 8–11):

8 $$\ast + {\mathrm{OH}}^- \to \ast {\mathrm{OH}} + {{e}}^-:\Delta G_1 = \Delta {{G}}_{{\mathrm{OH}}} - {\mathrm{eU}} + {{k}}_{\mathrm{b}}{\mathrm{Tln}}\left( {{{a}}_{{\mathrm{H}} + }} \right)$$

9 $$\ast {\mathrm{OH}} + {\mathrm{OH}}^- \to \ast {\mathrm{O}} + {\mathrm{H}}_2{\mathrm{O}}\left( {\mathrm{l}} \right) + {\mathrm{e}}^-:\Delta {{G}}_2 = \Delta {{G}}_{\mathrm{O}} - \Delta {{G}}_{{\mathrm{OH}}} - {\mathrm{eU}} + {{k}}_{\mathrm{b}}{\mathrm{Tln}}\left( {{{a}}_{{\mathrm{H}} + }} \right)$$

10 $$\ast {\mathrm{O}} + {\mathrm{OH}}^- \to \ast {\mathrm{OOH}} + {{e}}^-:\Delta {{G}}_3 = \Delta {{G}}_{{\mathrm{OOH}}} - \Delta {{G}}_{\mathrm{O}} - {\mathrm{eU}} + {{k}}_{\mathrm{b}}{\mathrm{Tln}}\left( {{{a}}_{{\mathrm{H}} + }} \right)$$

11 $$\ast {\mathrm{OOH}} + {\mathrm{OH}}^- \to {\mathrm{O}}_2\left( {\mathrm{g}} \right) + {\mathrm{H}}_2{\mathrm{O}}\left( {\mathrm{l}} \right) + {{e}}^-:\Delta {{G}}_4 = 4.92\left[ {{\mathrm{eV}}} \right] - \Delta {{G}}_{{\mathrm{OOH}}} - {\mathrm{eU}} + {{k}}_{\mathrm{b}}{\mathrm{Tln}}\left( {{{a}}_{{\mathrm{H}} + }} \right)$$

where ∆*G*_O_, ∆*G*_OH_, and ∆*G*_OOH_ are the Gibbs free energies of *O, *OH, and *OOH intermediate reactions, respectively. Corrections to the Gibbs free energies were adopted from ref. ^13^. Theoretical overpotentials of all considered structures were calculated at the given pH by the following equation^49,50^: *η*^theory^ = max[∆*G*_1_, ∆*G*_2_, ∆*G*_3_, ∆*G*_4_]/*e* − 1.23 [*V*], whereas the pH effect is canceled out in evaluating the overpotential.

## Supplementary information

Supplementary information

Supplementary Video 1

Source data

## Data Availability

The data that support the plots within this paper and other findings of this study are available from the corresponding author upon reasonable request. The source data underlying Figs. 1a, c, 2e, f, 3a–f, and 4a, c–e, and Supplementary Figs. 6a, b, 7a, b, 9a, b, 10, 11a, 12a, b, 13a, b, 19a, b, 20a–c, 21a–d, 22a–d, 23, 24a, b, 29, and 30–37 are provided as a Source Data file.

## References

[1] Park J (2018). Hollow nanoparticles as emerging electrocatalysts for renewable energy conversion reactions. Chem. Soc. Rev..

[2] Roy C (2018). Impact of nanoparticle size and lattice oxygen on water oxidation on NiFeOxHy. Nat. Catal..

[3] Guan J (2018). Water oxidation on a mononuclear manganese heterogeneous catalyst. Nat. Catal..

[4] Tiwari JN (2018). Multicomponent electrocatalyst with ultralow Pt loading and high hydrogen evolution activity. Nat. Energy.

[5] Hunter BM, Gray HB, Müller AM (2016). Earth-abundant heterogeneous water oxidation catalysts. Chem. Rev..

[6] Tiwari JN (2019). High-performance hydrogen evolution by Ru single atoms and nitrided-Ru nanoparticles implanted on N-doped graphitic sheet. Adv. Energy Mater..

[7] Suen NT (2017). Electrocatalysis for the oxygen evolution reaction: recent development and future perspectives. Chem. Soc. Rev..

[8] Luo J (2014). Water photolysis at 12.3% efficiency via perovskite photovoltaics and Earth-abundant catalysts. Science.

[9] Vij V (2017). Nickel-based electrocatalysts for energy-related applications: oxygen reduction, oxygen evolution, and hydrogen evolution reactions. ACS Catal..

[10] Fan K (2016). Nickel–vanadium monolayer double hydroxide for efficient electrochemical water oxidation. Nat. Commun..

[11] Sultan S (2019). Single atoms and clusters based nanomaterials for hydrogen evolution, oxygen evolution reactions, and Full Water Splitting. Adv. Energy Mater..

[12] She ZW (2017). Combining theory and experiment in electrocatalysis: Insights into materials design. Science.

[13] Zhang B (2016). Homogeneously dispersed multimetal oxygen-evolving catalysts. Science.

[14] Jiang J (2017). Highly active and durable electrocatalytic water oxidation by a NiB0.45/NiOx core-shell heterostructured nanoparticulate film. Nano Energy.

[15] Kanan MW, Nocera DG (2008). In situ formation of an oxygen-evolving catalyst in neutral water containing phosphate and Co<sup>2+</sup&gt. Science.

[16] Yuan C-Z (2016). Cobalt phosphate nanoparticles decorated with nitrogen-doped carbon layers as highly active and stable electrocatalysts for the oxygen evolution reaction. J. Mater. Chem. A.

[17] Liu K (2018). High-performance transition metal phosphide alloy catalyst for oxygen evolution reaction. ACS Nano.

[18] Padhi AK, Nanjundaswamy KS, Masquelier C, Okada S, Goodenough JB (1997). Effect of structure on the Fe^3+^/Fe^2+^ couple in iron phosphates. J. Electrochem. Soc..

[19] Wen Y (2014). Expanded graphite as superior anode for sodium-ion batteries. Nat. Commun..

[20] Tiwari JN (2017). High-affinity-assisted nanoscale alloys as remarkable bifunctional catalyst for alcohol oxidation and oxygen reduction reactions. ACS Nano.

[21] Georgakilas V, Perman JA, Tucek J, Zboril R (2015). Broad family of carbon nanoallotropes: classification, chemistry, and applications of fullerenes, carbon dots, nanotubes, graphene, nanodiamonds, and combined superstructures. Chem. Rev..

[22] Fei H (2018). General synthesis and definitive structural identification of MN_4_C_4_ single-atom catalysts with tunable electrocatalytic activities. Nat. Catal..

[23] Sultan S (2018). Highly efficient oxygen reduction reaction activity of graphitic tube encapsulating nitrided Co_x_Fe_y_ alloy. Adv. Energy Mater..

[24] Fabbri E (2017). Dynamic surface self-reconstruction is the key of highly active perovskite nano-electrocatalysts for water splitting. Nat. Mater..

[25] Li H (2018). Synergetic interaction between neighbouring platinum monomers in CO_2_ hydrogenation. Nat. Nanotechnol..

[26] Bunău O, Joly Y (2009). Self-consistent aspects of x-ray absorption calculations. J. Phys. Condens. Matter.

[27] Bourke JD, Chantler CT, Joly Y (2016). FDMX: extended X-ray absorption fine structure calculations using the finite difference method. J. Synchrotron Radiat..

[28] Cobo S (2012). A Janus cobalt-based catalytic material for electro-splitting of water. Nat. Mater..

[29] Liu W (2016). A highly active and stable hydrogen evolution catalyst based on pyrite-structured cobalt phosphosulfide. Nat. Commun..

[30] Jiao L, Zhou Y-X, Jiang H-L (2016). Metal–organic framework-based CoP/reduced graphene oxide: high-performance bifunctional electrocatalyst for overall water splitting. Chem. Sci..

[31] You B, Sun Y (2016). Hierarchically porous nickel sulfide multifunctional superstructures. Adv. Energy Mater..

[32] Xu X, Song F, Hu X (2016). A nickel iron diselenide-derived efficient oxygen-evolution catalyst. Nat. Commun..

[33] Amin BG, Swesi AT, Masud J, Nath M (2017). CoNi_2_Se_4_ as an efficient bifunctional electrocatalyst for overall water splitting. Chem. Commun..

[34] Zhang P (2018). Dendritic core-shell nickel-iron-copper metal/metal oxide electrode for efficient electrocatalytic water oxidation. Nat. Commun..

[35] Ng JWD (2016). Gold-supported cerium-doped NiOx catalysts for water oxidation. Nat. Energy.

[36] Hou Y (2011). Bioinspired molecular co-catalysts bonded to a silicon photocathode for solar hydrogen evolution. Nat. Mater..

[37] Zhang R (2018). Engineering cobalt defects in cobalt oxide for highly efficient electrocatalytic oxygen evolution. ACS Catal..

[38] Stevens MB, Trang CDM, Enman LJ, Deng J, Boettcher SW (2017). Reactive Fe-sites in Ni/Fe (oxy)hydroxide are responsible for exceptional oxygen electrocatalysis activity. J. Am. Chem. Soc..

[39] Sun. W (2018). Effect of lattice strain on the electro-catalytic activity of IrO_2_ for water splitting. Chem. Commun..

[40] Xiong D, Wang X, Li W, Liu L (2016). Facile synthesis of iron phosphide nanorods for efficient and durable electrochemical oxygen evolution. Chem. Commun..

[41] Zhang G (2016). Highly active and stable catalysts of phytic acid-derivative transition metal phosphides for full water splitting. J. Am. Chem. Soc..

[42] Pan Y (2018). Core–shell ZIF-8@ZIF-67-derived CoP nanoparticle-embedded N-doped carbon nanotube hollow polyhedron for efficient overall water splitting. J. Am. Chem. Soc..

[43] Ravel B, Newville M (2005). ATHENA, ARTEMIS, HEPHAESTUS: data analysis for X-ray absorption spectroscopy using IFEFFIT. J. Synchrotron Radiat..

[44] Kresse G, Furthmüller J (1996). Efficient iterative schemes for ab initio total-energy calculations using a plane-wave basis set. Phys. Rev. B.

[45] Blöchl PE (1994). Projector augmented-wave method. Phys. Rev. B.

[46] Kresse G, Joubert D (1999). From ultrasoft pseudopotentials to the projector augmented-wave method. Phys. Rev. B.

[47] Perdew JP, Burke K, Ernzerhof M (1996). Generalized gradient approximation made simple. Phys. Rev. Lett..

[48] Tkatchenko A, Scheffler M (2009). Accurate molecular van der waals interactions from ground-state electron density and free-atom reference data. Phys. Rev. Lett..

[49] Rossmeisl J, Logadottir A, Nørskov JK (2005). Electrolysis of water on (oxidized) metal surfaces. Chem. Phys..

[50] Man IC (2011). Universality in oxygen evolution electrocatalysis on oxide surfaces. ChemCatChem.

