# Supplementary material for: Superb water splitting activity of the electrocatalyst Fe3Co(PO4)4 designed with computation aid
Source: Nat Commun. 2019 Nov 15;10:5195. doi: 10.1038/s41467-019-13050-3 (PMC6858335; doi:10.1038/s41467-019-13050-3)
Supplement: Supplementary file 1 — Supplementary information [file 41467_2019_13050_MOESM1_ESM.pdf]

SUPPLEMENTARY INFORMATION

Superb water splitting activity of the electrocatalyst $Fe_3Co(PO_4)_4$ designed with computation-aid

Siraj Sultan[1$], Miran Ha[1,2$], Dong Yeon Kim[1$], Jitendra N. Tiwari[1*], Chang Woo Myung,[1*] Abhishek Meena[1], Tae Joo Shin[3], Keun Hwa Chae[4] & Kwang S. Kim[1*]

[1]Center for Superfunctional Materials, Department of Chemistry, Ulsan National Institute of Science and Technology (UNIST), 50 UNIST-gil, Ulsan 689-798, Korea

[2]Department of Energy and Chemical Engineering, UNIST, Ulsan, Korea

[3]UNIST Central Research Facilities, UNIST, Ulsan, Korea.

[4]Advanced Analysis Center, Korea Institute of Science and Technology, 5 Hwarangno 14-gil, Seongbuk-gu, Seoul 02792, Korea.

[$]*These authors contributed equally to this work.*

*Correspondence and requests for materials should be addressed to J.N.T. (Email: jitendra@unist.ac.kr), C.W.M. (cwmyung@unist.ac.kr) or K.S.K. (Email: kimks@unist.ac.kr).

Supplementary Figures

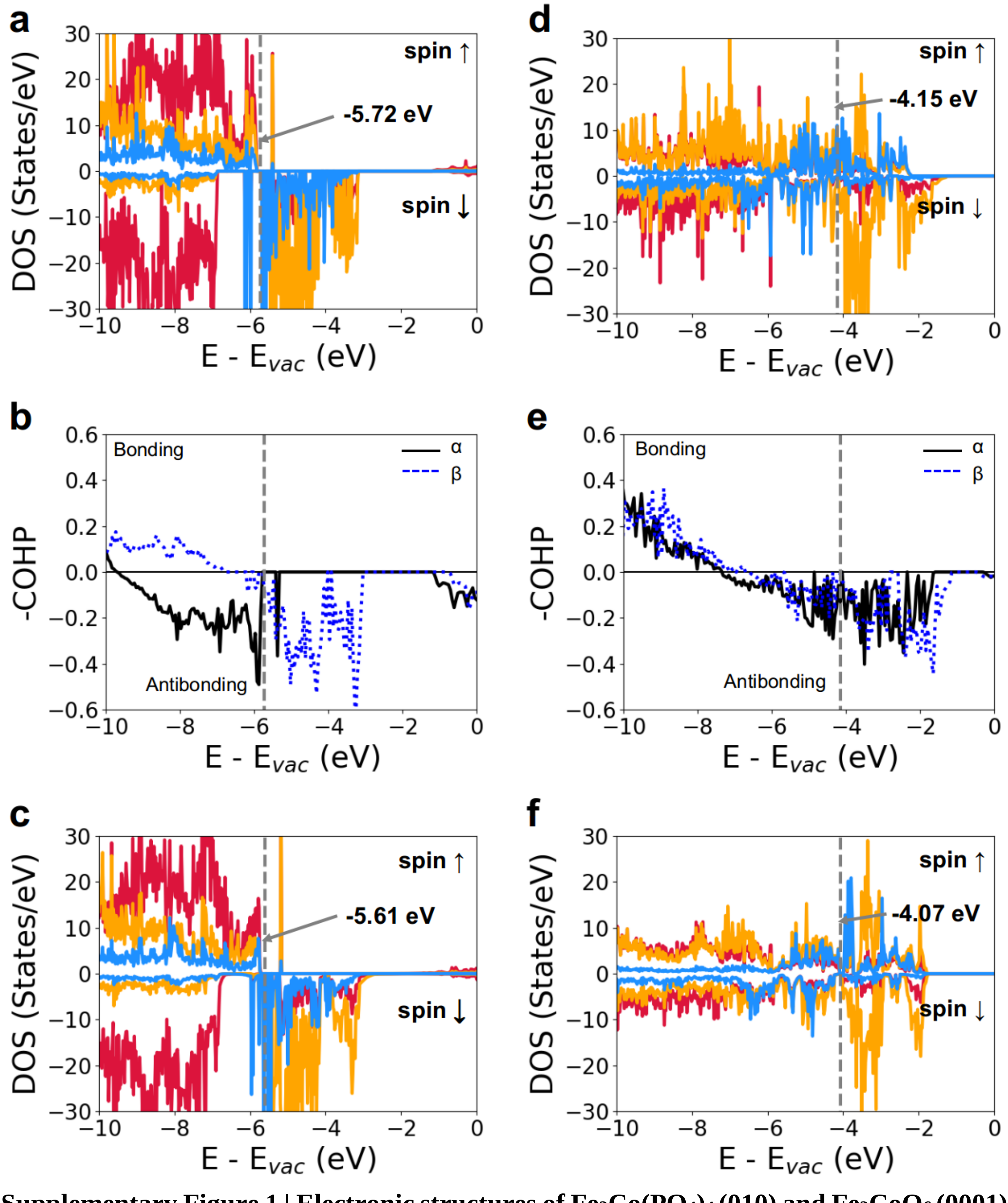

Supplementary Figure 1 | Electronic structures of $Fe_3Co(PO_4)_4$ (010) and Fe_3CoO_6 (0001). (a, d) Partial density of states (PDOS) and (b, e) crystal orbital Hamilton population (COHP)[1, 2, 3, 4] of M-O (M=Fe, Co) bonds in $Fe_3Co(PO_4)_4$ (010) (a, b) and Fe_3CoO_6 (0001) (d, e) with respect to energy (versus vacuum), which clearly demonstrate the effect of phosphate substitution. Because of the stabilization of M-O antibonding states, there exists a pronounced energy downshift of $Fe_3Co(PO_4)_4$(010) as compared to Fe_3CoO_6(0001). The effect of reduced graphene oxide (rGO) on electronic properties of both surfaces is small based on similar PDOS of (c) $Fe_3Co(PO_4)_4$(010)/rGO and (f) Fe_3CoO_6(0001)/rGO with PDOS of (a) and (d), respectively. Fe: orange, Co: skyblue, O: red.

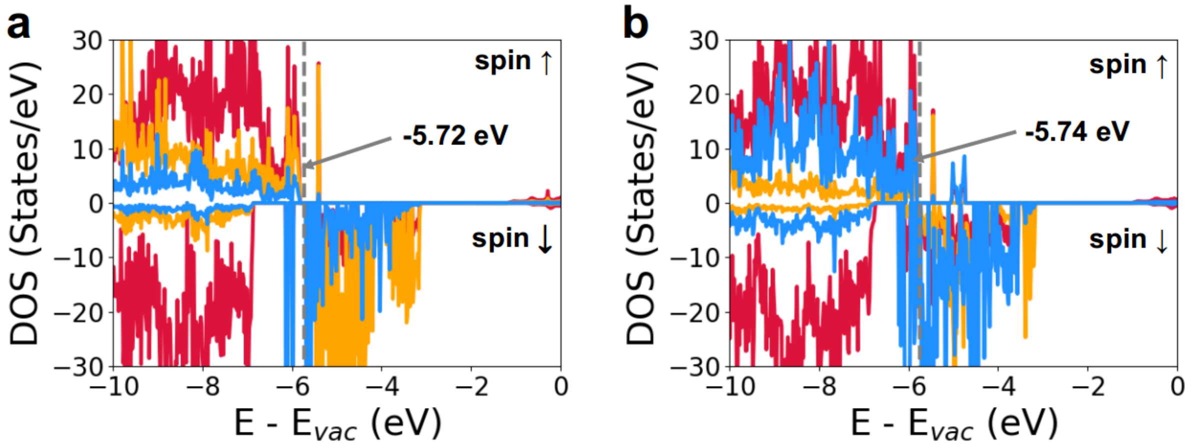

Supplementary Figure 2 | Partial density of states (PDOS) of (a) $Fe_3Co(PO_4)_4$ (010) and (b) $FeCo_3(PO_4)_4$ (010). Antibonding states of $FeCo_3(PO_4)_4$ downshifted as compared to $Fe_3Co(PO_4)_4$ (010). Fe: orange, Co: skyblue, O: red.

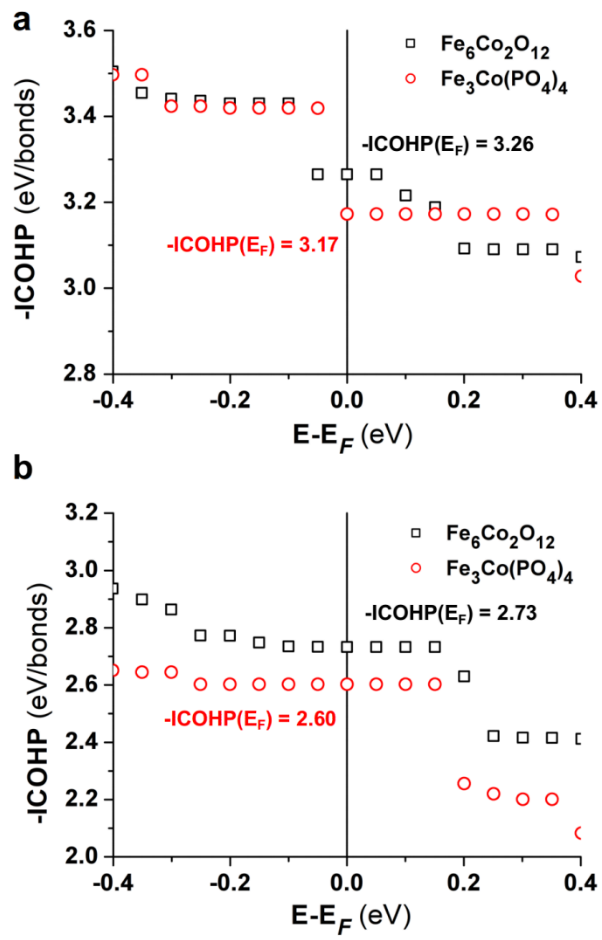

Supplementary Figure 3 | Integrated crystal orbital Hamilton population (ICOHP) of $Fe_3Co(PO_4)_4$ and $Fe_6Co_2O_{12}$ for (a) Fe-*O and (b) Co-*O. The ICOHP up to Fermi energy (ICOHP(E_F)) is related to bond strength (More positive -ICOHP implies stronger bond). In both Fe and Co sites, M-O (M=Fe, Co) of $Fe_3Co(PO_4)_4$ is weaker than that of $Fe_6Co_2O_{12}$.

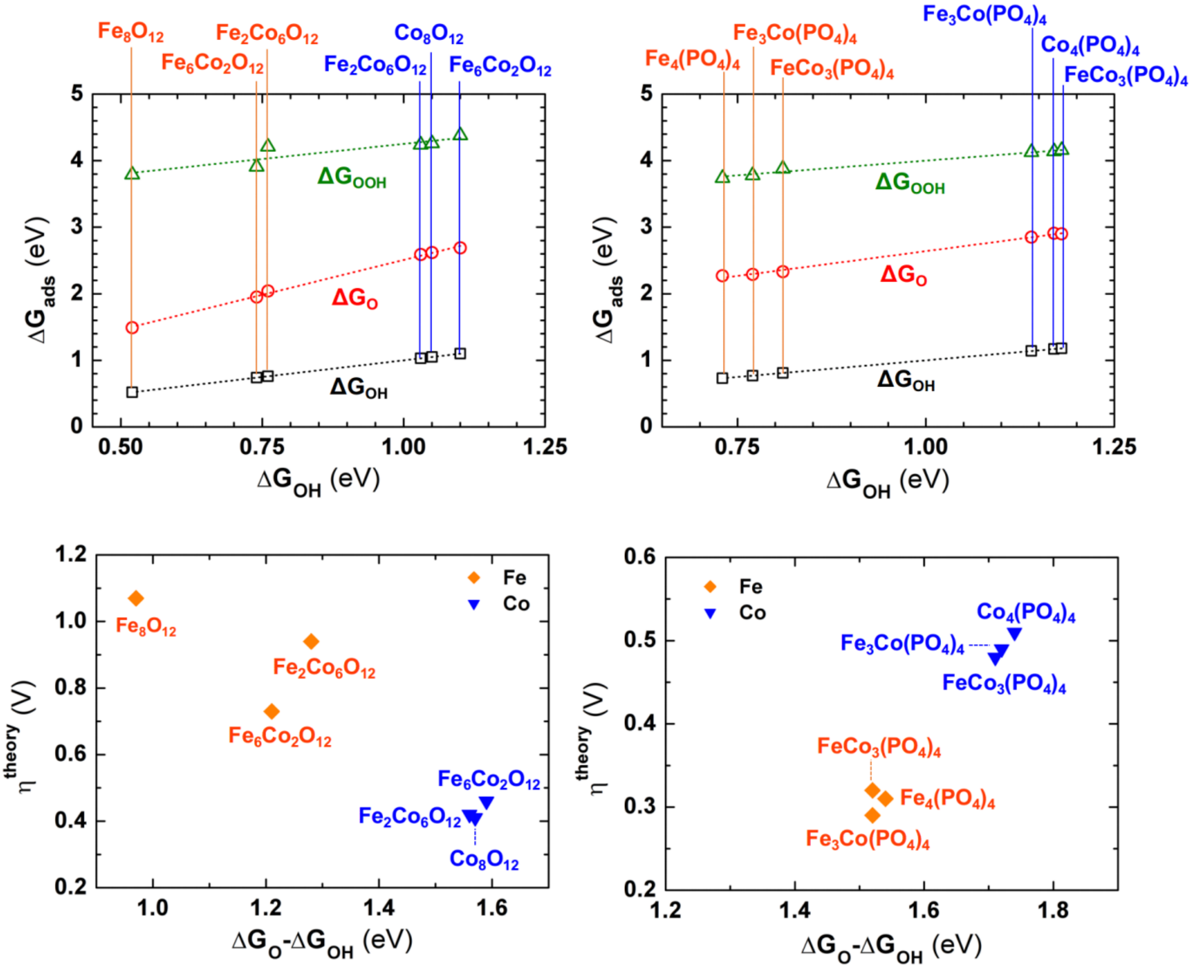

Supplementary Figure 4 | Adsorption free energies for OER intermediates versus ΔG_{OH} and theoretical overpotential (η^{theory}) versus ΔG_O-ΔG_{OH}. Adsorption Free energies of *O, *OH and *OOH (ΔG_O, ΔG_{OH} and ΔG_{OOH}, respectively) and η^{theory} at Fe-sites (orange) and Co-sites (blue) of Fe_8O_{12}, $Fe_6Co_2O_{12}$, $Fe_2Co_6O_{12}$, Co_8O_{12}, $Fe_4(PO_4)_4$, $Fe_3Co(PO_4)_4$, $FeCo_3(PO_4)_4$, and $Co_4(PO_4)_4$ clusters are plotted as a function of ΔG_{OH} and ΔG_O-ΔG_{OH}, respectively. Free energies for all models are listed in **Supplementary Table 1**.

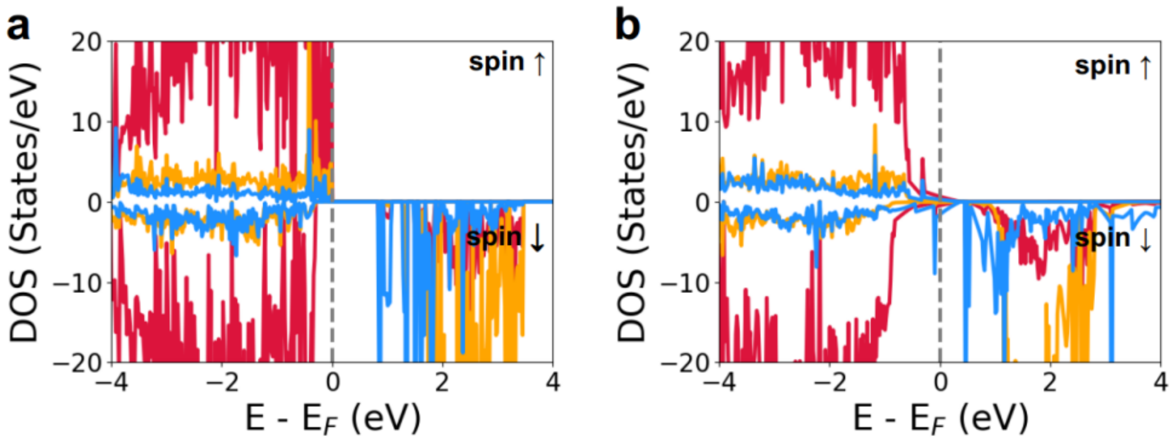

Supplementary Figure 5 | Partial density of states (PDOS) of (a) $Fe_3Co(PO_4)_4$(010) and (b) $Fe_3Co_3(PO_4)_4$(010)/rGO using PBE+U (U_{eff} (Fe) = 4 eV, U_{eff} (Co) = 3.3 eV). The rGO support makes metal phosphates conductive. Fe: orange, Co: skyblue, O: red.

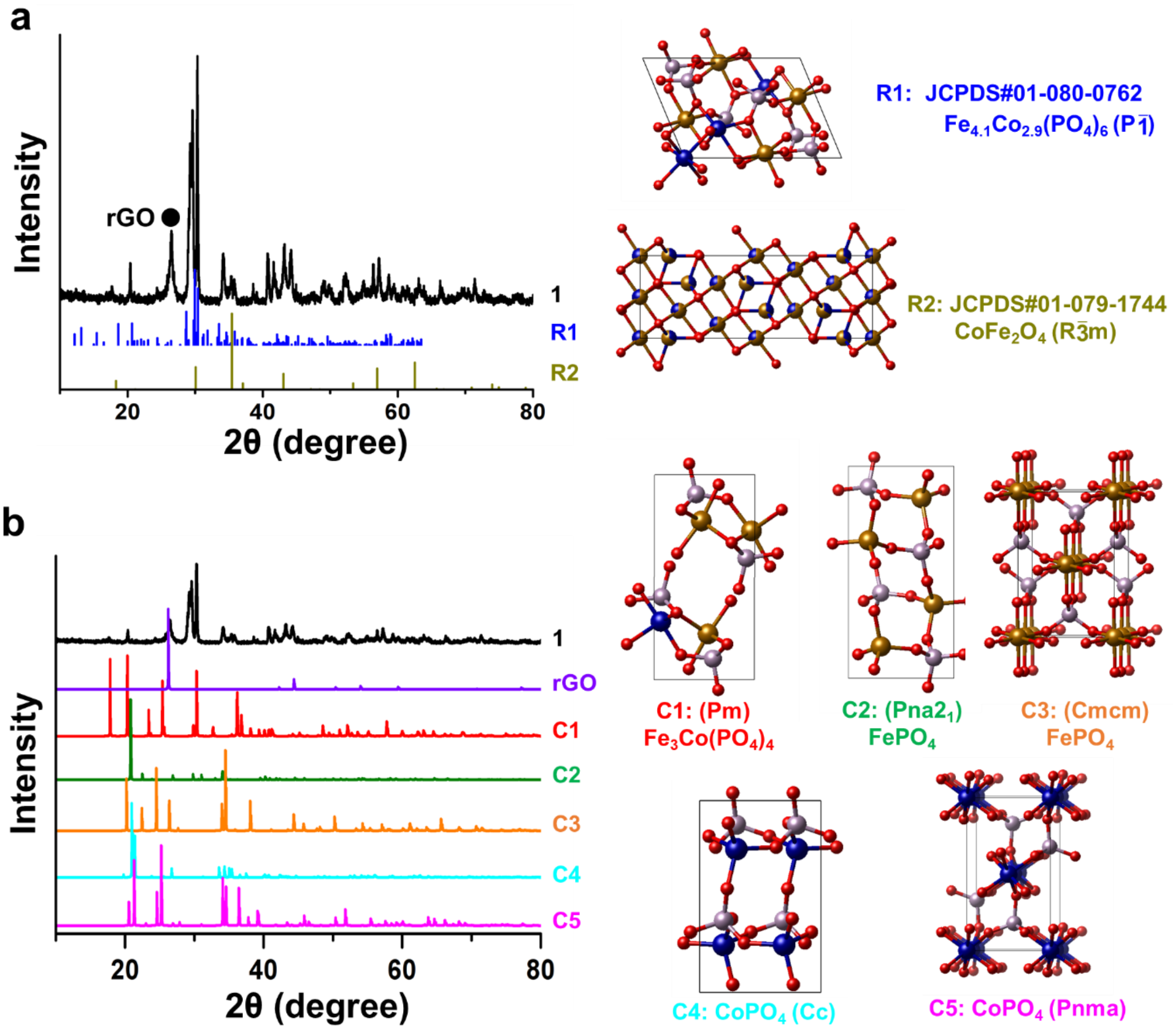

Supplementary Figure 6 | X−ray diffraction (XRD) characterization for 1 [($Fe_3Co(PO_4)_4$@rGO] compared with the experimental XRD spectra [obtained from crystal structures of Reference [5]: $Fe_{4.1}Co_{2.9}(PO_4)_4$ and Reference 2: $CoFe_2O_4$] and the calculated XRD spectra [based on different metal phosphates crystal structures]**.** (a) Comparison of the experimental XRD spectra of **1**. (b) Comparison of the experimental XRD spectra of **1** with the calculated XRD spectra (C1-C5) based on DFT-predicted various metal phosphates crystal structures. The XRD spectra of **1** are similar mainly to those of C1: $Fe_3Co(PO_4)_4$ (Pm) and partly to those of C3: $FePO_4$ (Cmcm).

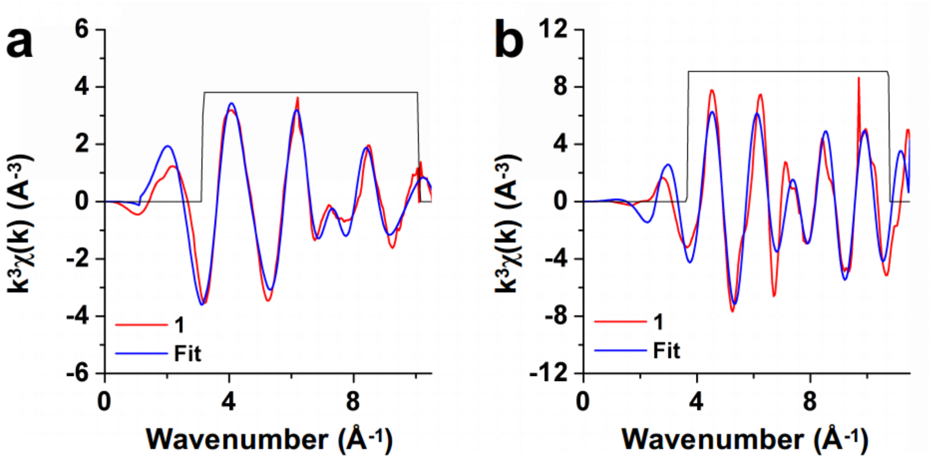

Supplementary Figure 7 | EXAFS χ(k) signals in k-space and the corresponding least-squares fit (black solid line) for 1st shell (a) Fe and (b) Co. **1**: $Fe_3Co(PO_4)_4$@rGO.

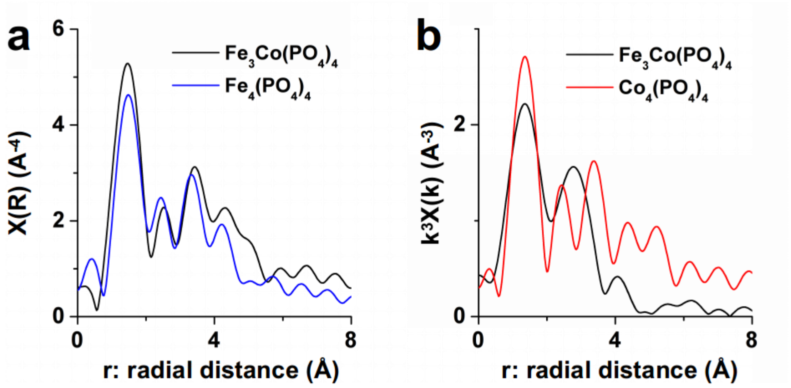

Supplementary Figure 8 | Calculated FT-EXAFS spectra in r-space of $Fe_3Co(PO_4)_4$ crystal structure compared with Fe_4 $(PO_4)_4$ and Co_4 $(PO_4)_4$. (a) Fe K-edge XAFS and (b) Co site K-edge XAFS are calculated using FDMX[6, 7] with a full-potential finite difference method (FDM). Both Fe K-edge XAFS of Fe_4 $(PO_4)_4$ and Co site K-edge XAFS of $Co_4(PO_4)_4$ are similar. Though Fe K-edge XAFS of $Fe_3Co(PO_4)_4$ is similar to that of $Fe_4(PO_4)_4$, the Co K-edge XAFS of $Fe_3Co(PO_4)_4$ is significantly different from that of $Co_4(PO_4)_4$, because the former has no neighboring Co atoms, while $Co_4(PO_4)_4$ has four neighboring Co atoms.

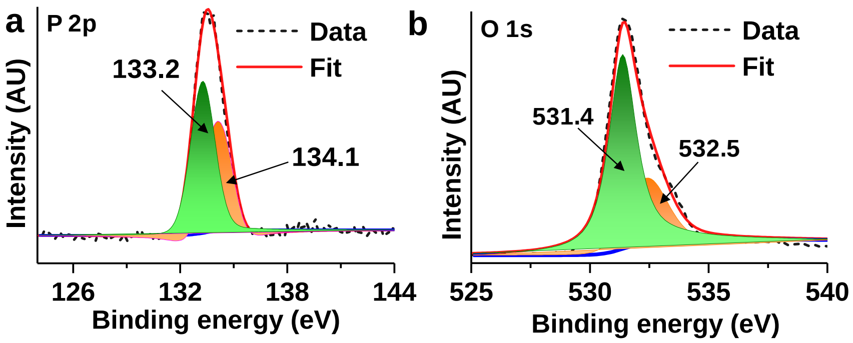

Supplementary Figure 9 | X-ray Photoelectron Spectroscopy (XPS) core-level spectra of 1 before stability tests. a, P_{2p}, and **b,** O_{1s}.

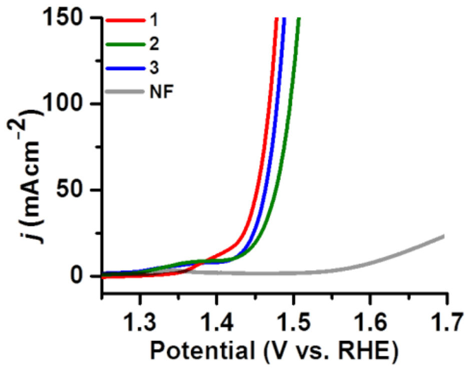

Supplementary Figure 10 | OER performance. **1:**($(Fe_3Co(PO_4)_4$@rGO)@NF, **2:**($FeCo(PO_4)_2$@rGO)@NF, **3:**($Fe_2Co(PO_4)_3$@rGO)@NF and **NF** in 1M KOH aqueous solution. NF: Nickel foam.

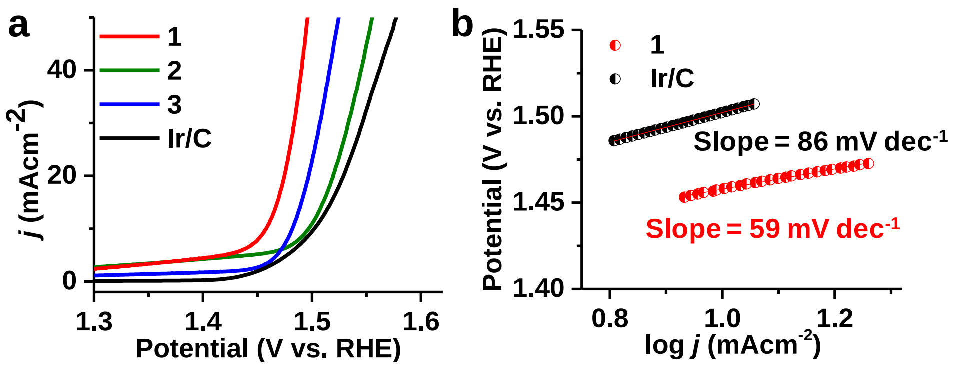

Supplementary Figure 11 | OER performance and Tafel slope. a, 1:($Fe_3Co(PO_4)_4$@rGO)@GCE, **2:**($FeCo(PO_4)_2$@rGO)@GCE, **3:**($Fe_2Co(PO_4)_3$@rGO)@GCE, and Ir/C@GCE in 1M KOH aqueous solution. GCE: Glassy carbon electrode. **b,** OER Tafel plots of **1** and **Ir/C** catalysts derived from a.

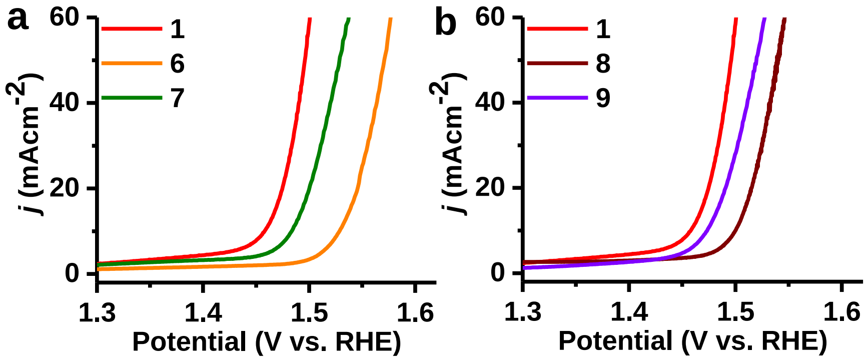

Supplementary Figure 12 | Effect of GO and red phosphorous on the OER performance. a, effect of GO amounts on the OER performance. 6 [0.7 g $FeCl_3$, 0.35 g $Co(ClO_4)_2.6H_2O$, 100 mg GO and 0.07g red phosphorus] and **7** [0.7 g $FeCl_3$, 0.35 g $Co(ClO_4)_2.6H_2O$, 300 mg GO and 0.07g red phosphorus]. **b,** effect of red phosphorous amounts on the OER performance **8** [0.7 g $FeCl_3$, 0.35 g $Co(ClO_4)_2.6H_2O$, 200 mg GO and 0.035g red phosphorus] and **9** [0.7 g $FeCl_3$, 0.35 g $Co(ClO_4)_2.6H_2O$, 200 mg GO and 0.140 g red phosphorus].

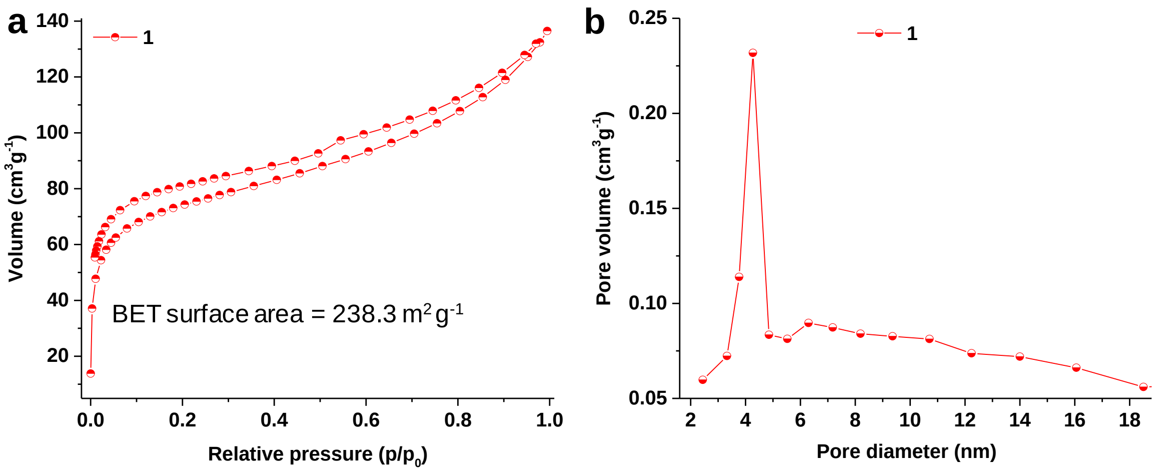

Supplementary Figure 13 | BET surface area analysis and pore size distribution 1. a, N_2 adsorption-desorption isotherm. **b**, pore size distribution from Barret–Joyner–Halenda (BJH) calculation.

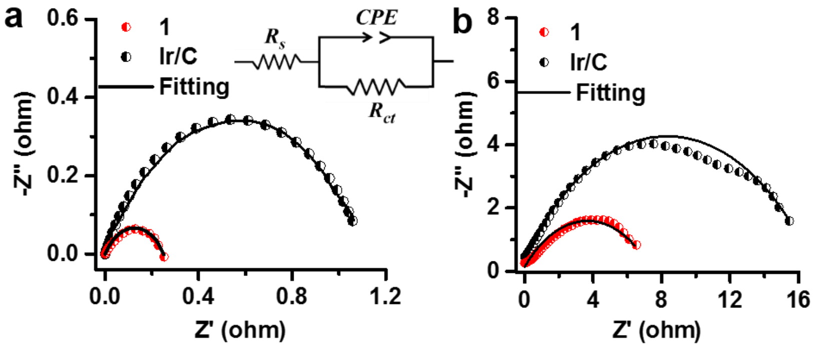

Supplementary Figure 14 | Impedance measurements. EIS Nyquist plots of 1 and Ir/C on nickel foam (a) and 1 and Ir/C on GCE substrate (b). In the Nyquist plots, an imaginary part (–*Z*'') and a real part (*Z*') of characteristic curves are plotted as *y* axis and *x* axis, respectively. The inset is the fitting equivalent circuit for the impedance spectra.

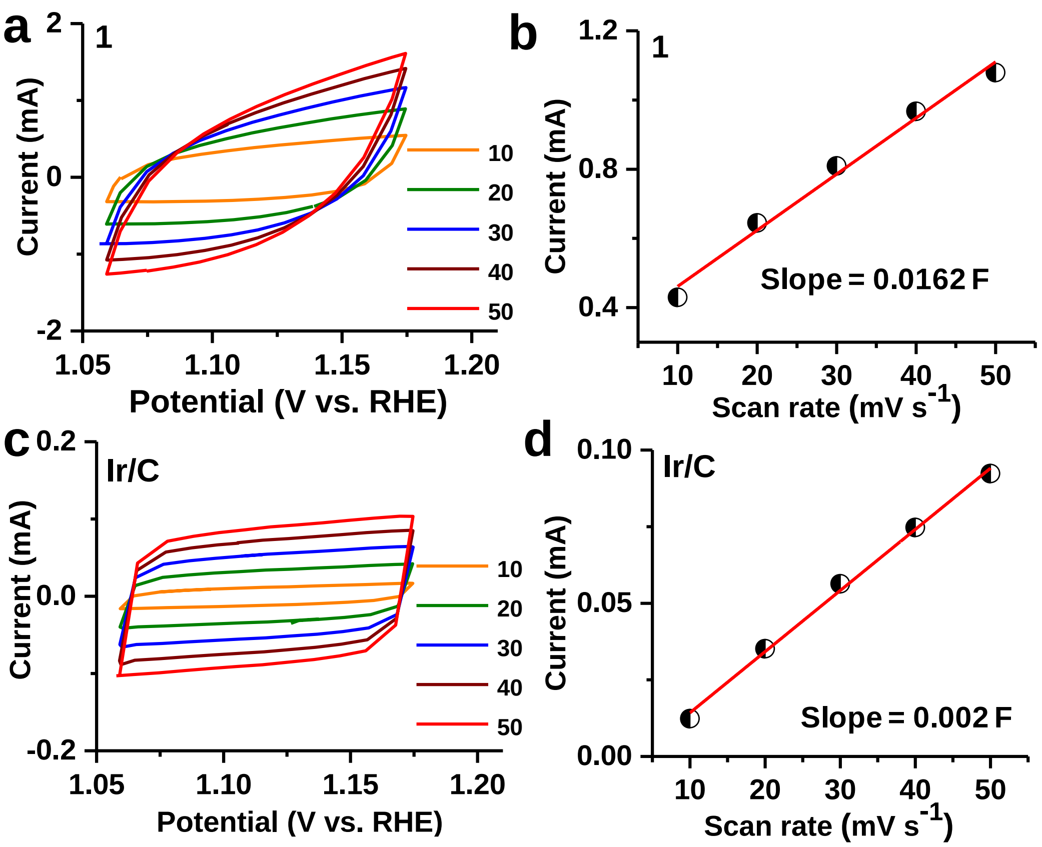

Supplementary Figure 15 | Double layer capacitance measurements. Cyclic voltammograms of **1** (a), and **Ir/C** (c) which were recorded at different scan rates in the voltage window of 1.059 to 1.174 V versus RHE. Scan rate dependence of the current densities of **1** (b) and **Ir/C** (d) at 1.125 V versus RHE.

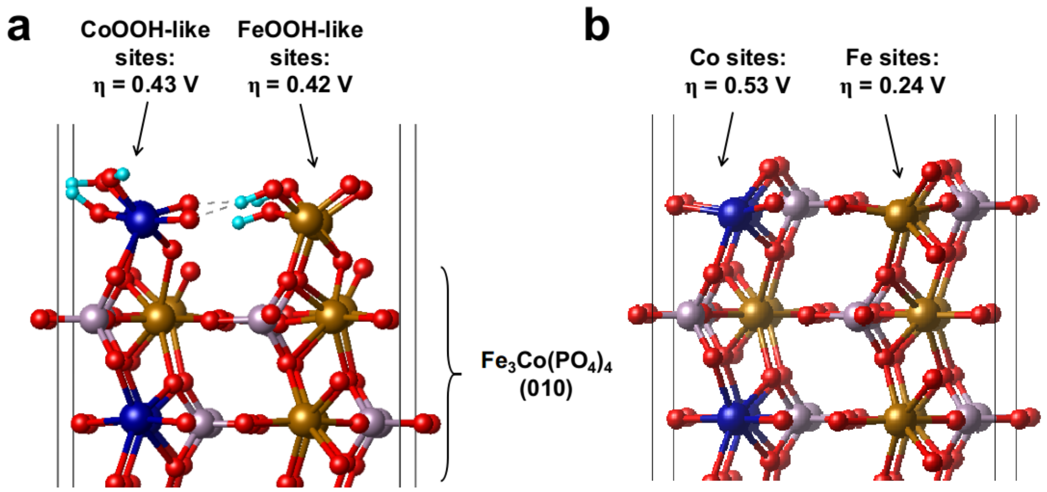

Supplementary Figure 16 | Structure and OER activity of (a) FeOOH- and CoOOH-like sites on top of $Fe_3Co(PO_4)_4$ (010) and (b) Fe and Co sites of $Fe_3Co(PO_4)_4$ (010). Fe: orange, Co: blue, O: red, H: cyan.

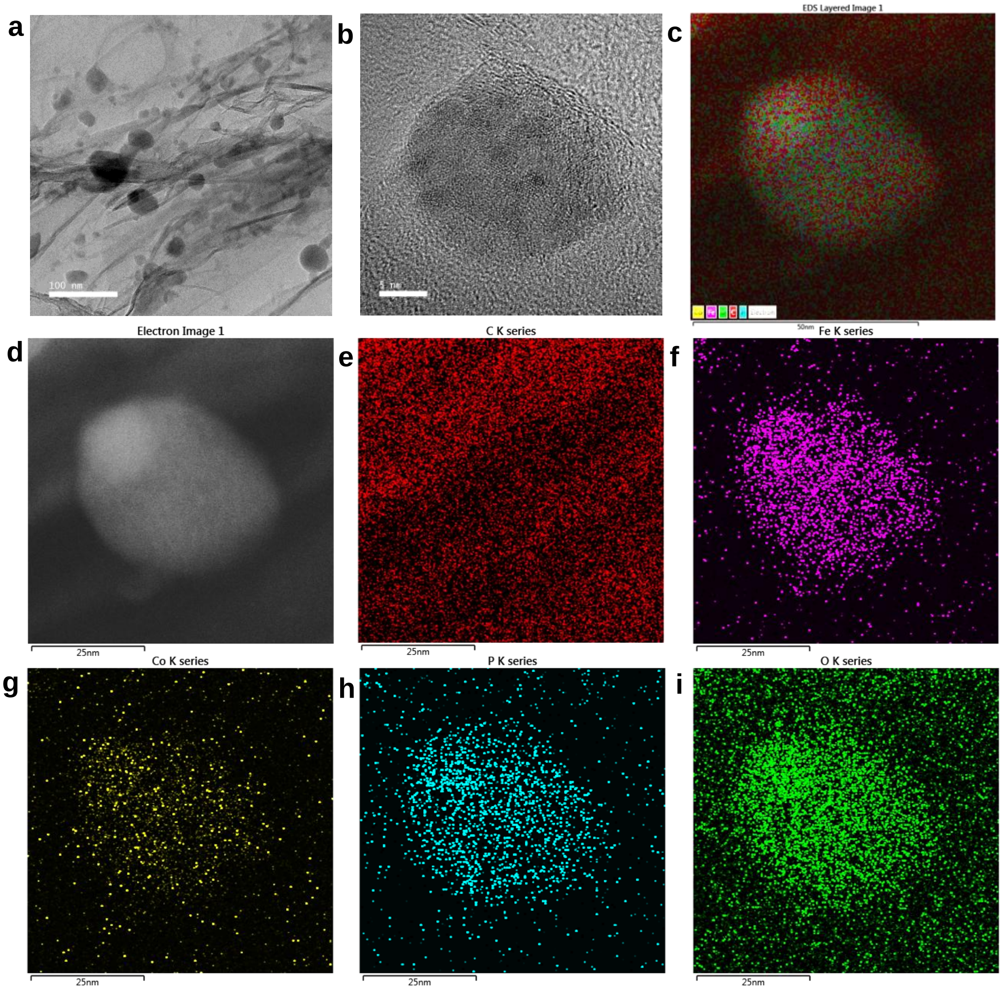

Supplementary Figure 17 | Microstructural characterization after 5000 CV cycles test. a, Low-magnification and **b,** High magnification TEM images. **c**, Overlapping image of d-i. **d-i,** STEM-HAADF image and elemental maps of C (e), Fe (f), Co (g), P (h), and O (i).

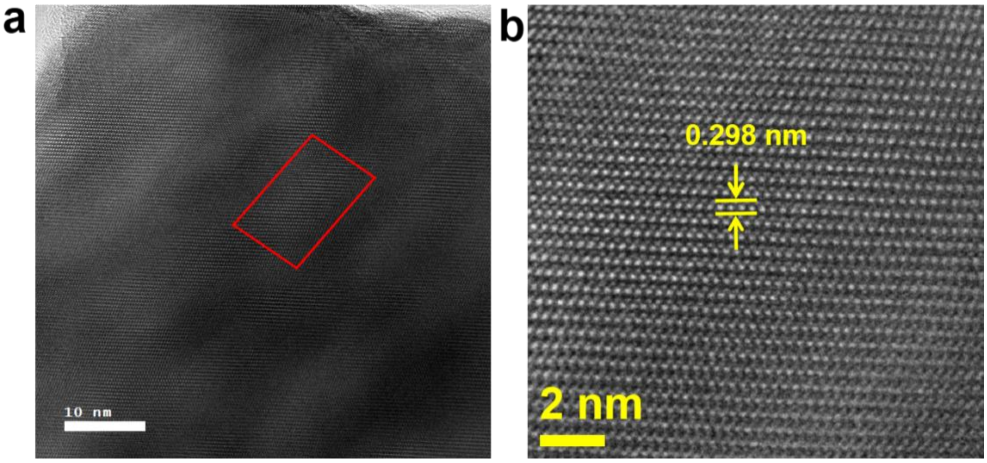

Supplementary Figure 18 | HRTEM images of 1 after 5000 CV cycles test, a, HRTEM image and **b,** Magnified HRTEM image taken from the selected area in **a**.

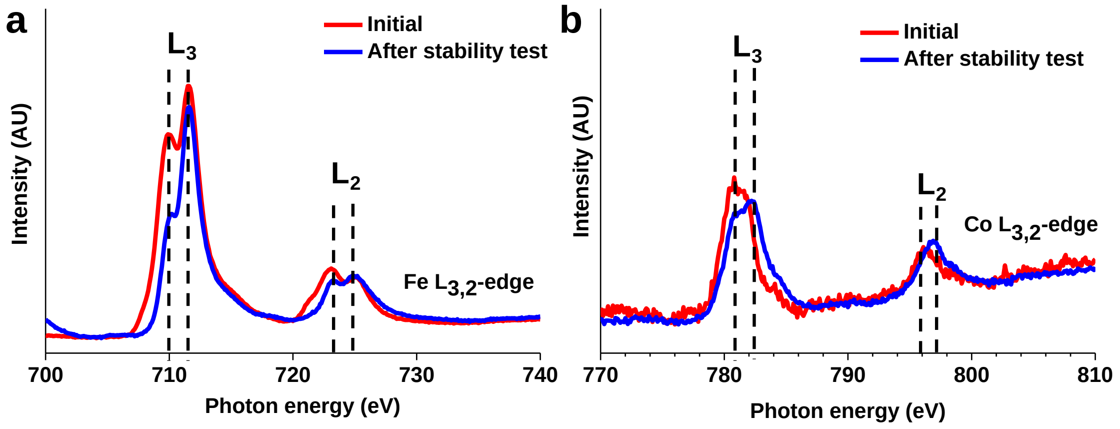

Supplementary Figure 19 | Comparison of XAS spectra of 1 before and after stability tests. a, Fe $L_{3,2}$-edge and **b,** Co $L_{3,2}$-edge. After a 5000-cycle test, the Fe $L_{3,2}$-edge XAS spectra for **1** indicates that the positions of the L_3 and L_2 peaks are almost same for both Fe and Co, suggesting that not only Fe^{2+} and Fe^{3+} states but also Co^{2+} and Co^{3+} states kept almost same during the cycling. Nevertheless, though very small, $L_{3,2}$-edge XAS spectra shifted very slightly to higher energy, indicating that Fe/Co is very slightly oxidized during the OER stability test. As a result, FeO_x/CoO_x or FeOOH/CoOOH could be slightly formed during the OER process as the reviewer pointed out. However, CoOOH is expected to be a less active site because of its higher overpotential than RuO_2 (which has a much lower activity than ours)[8] and its less inductive effect of P towards Co sites in our catalyst. Phosphate sites are not active, as the reviewer addressed. However, metal sites are active. The DFT calculations demonstrate that the Fe-sites of $Fe_3Co(PO_4)_4$ are active sites with overpotential 0.24 V in excellent agreement with the experiment.

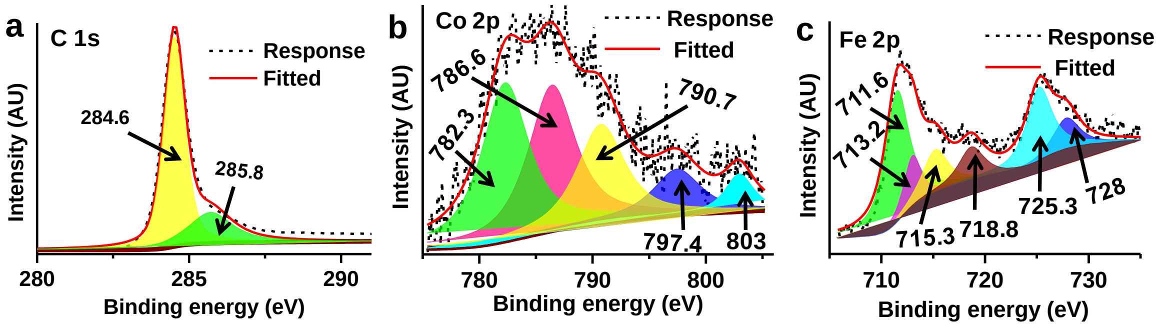

Supplementary Figure 20 | XPS core-level spectra of 1 before stability tests. a, C 1s; **b,** Co 2p; **c,** Fe 2p.

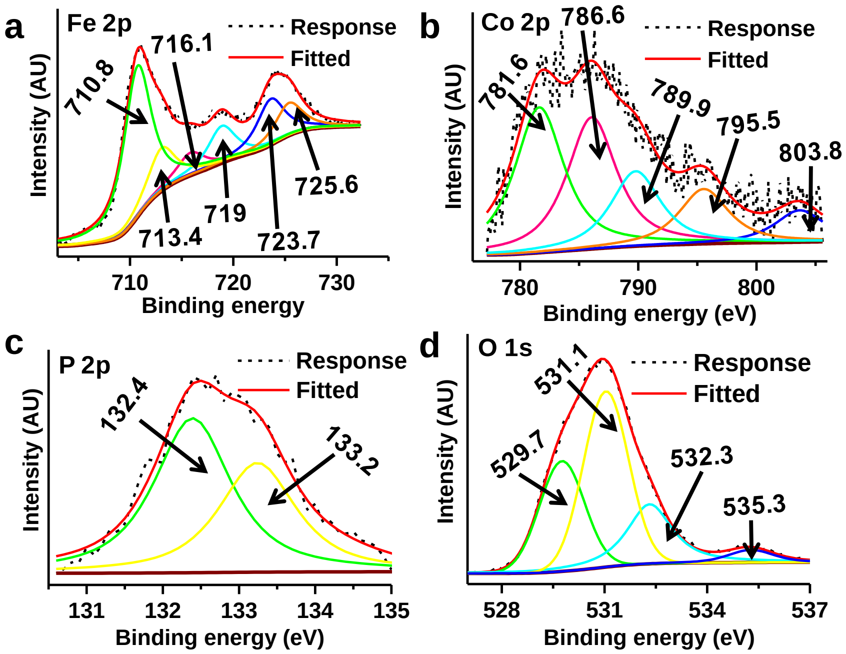

Supplementary Figure 21 | XPS core-level spectra of 1 after 5000 CV cycles stability test. **a,** Fe 2p; **b,** Co 2p; **c,** P 2p; **d,** O 1s. Post-mortem analysis to investigate the surface reaction effect during the OER process shows insignificant change in peak positions of Fe, Co, P, and O. However, O 1s shows the two characteristic peak at 529.7 and 535.3 eV, which are attributed to O^{2-} ions of the lattice oxygen[9] and surface hydroxyl/water moiety [10], respectively.

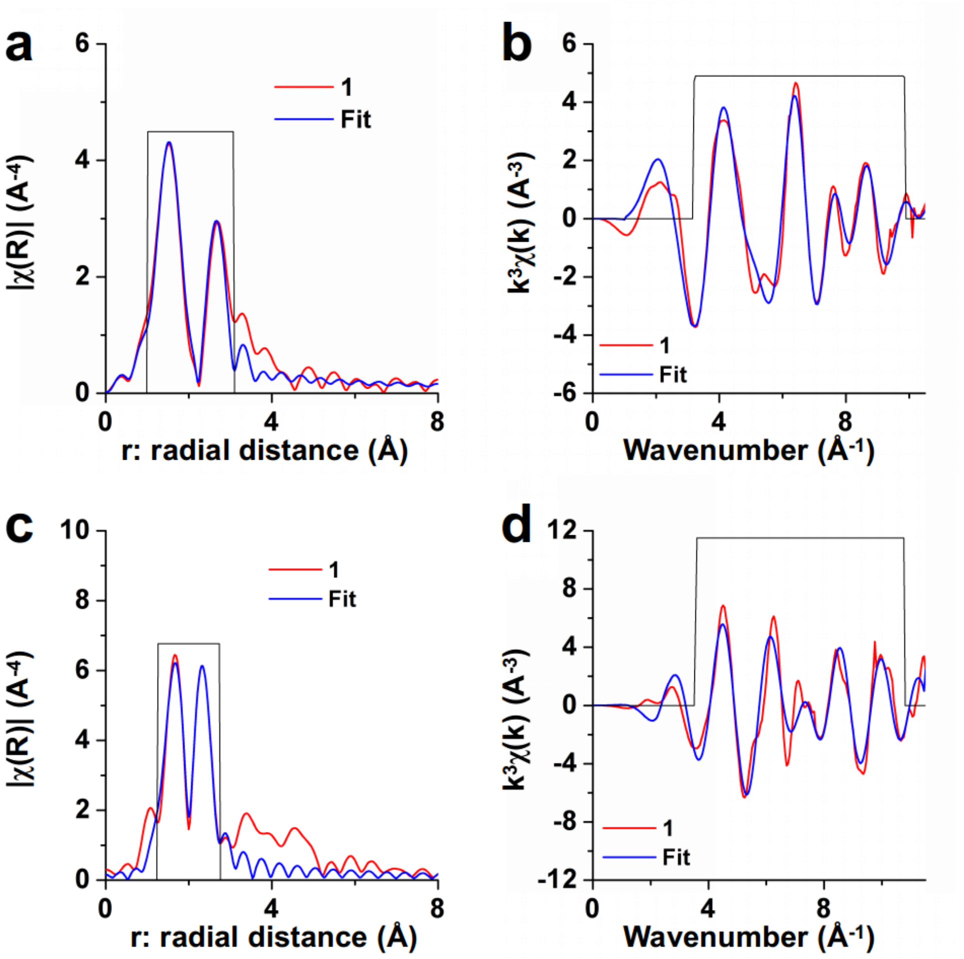

Supplementary Figure 22 | Curve-fitting analysis for (a,b) Fe-K edge and (c,d) Co K-edge EXAFS of $Fe_3Co(PO_4)_4$@rGO after OER test. (a,c) FT-EXAFS spectra in r-space and the corresponding least-squares fit for 1st and 2nd shells. (b,d) k^3 weighted EXAFS $\chi(k)$ in k-space and the corresponding least-squares fit for 1st and 2nd shells.

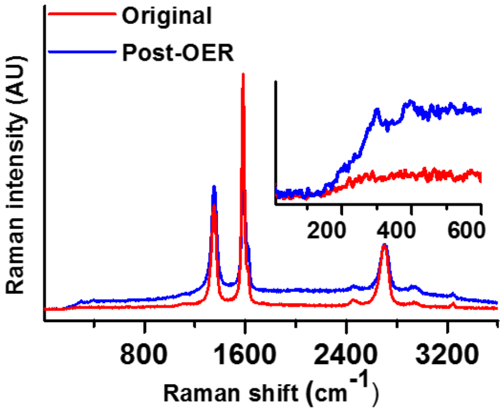

Supplementary Figure 23 | Raman analysis. Raman spectra of **1** before and after 5000 CV cycles stability test. Two small peak at 300 and 400 cm^{-1} were observed after OER stability, indicating the partly oxidization of Fe or Co on the $Fe_3Co(PO_4)_4$ surface.

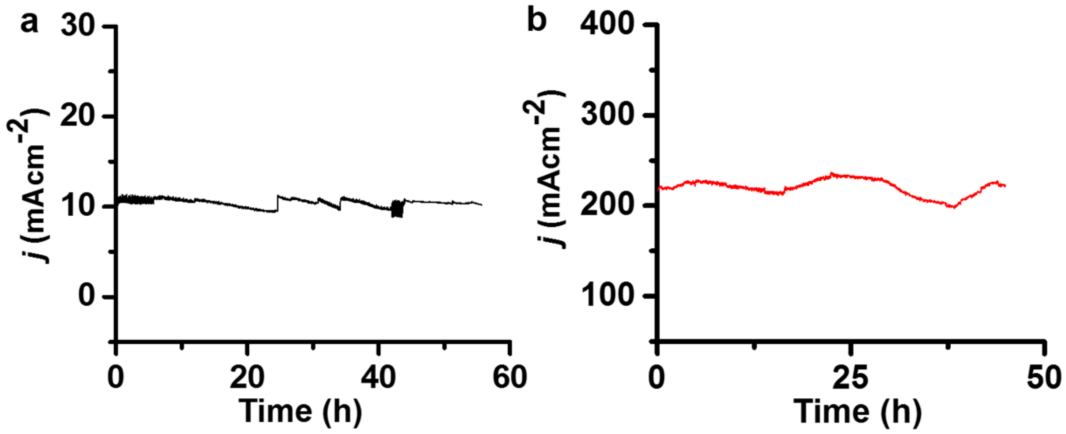

Supplementary Figure 24 | Chronoamperometric tests. a, Stability test of $Fe_3Co(PO_4)_4$@rGO catalysts loaded on glassy carbon electrode in 1M KOH. **b**, Stability test of $Fe_3Co(PO_4)_4$@rGO catalysts loaded on nickel foam in 1M KOH at high current density. (the area of nickel foam for this stability test was 1cm^2).

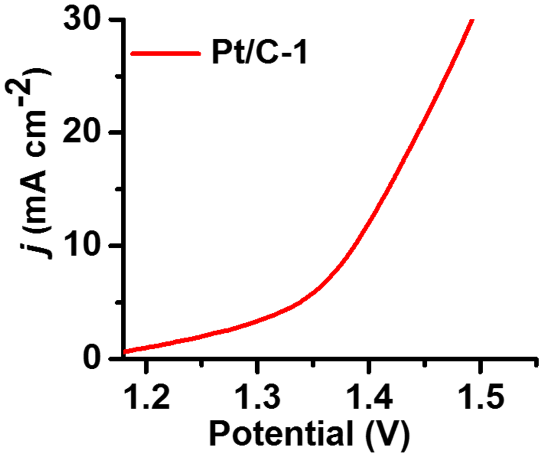

Supplementary Figure 25 | Whole water splitting. Current density of Pt/C-**1** for overall water splitting in 6M KOH solution. The catalyst loading at cathode and anode was 5 mg cm^{-2}.

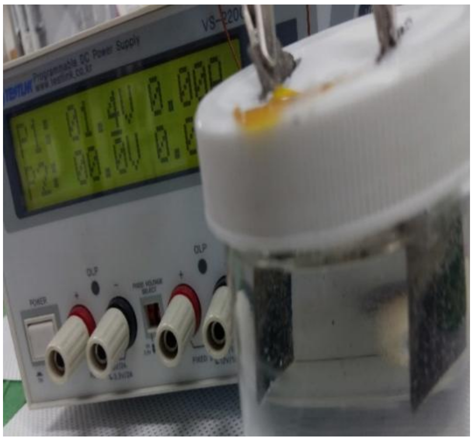

Supplementary Figure 26 | Photographic image of alkaline water splitting at 1.4 V in a two-electrode configuration for overall water splitting in 6M KOH solution, indicating the oxygen and hydrogen bubbles generation.

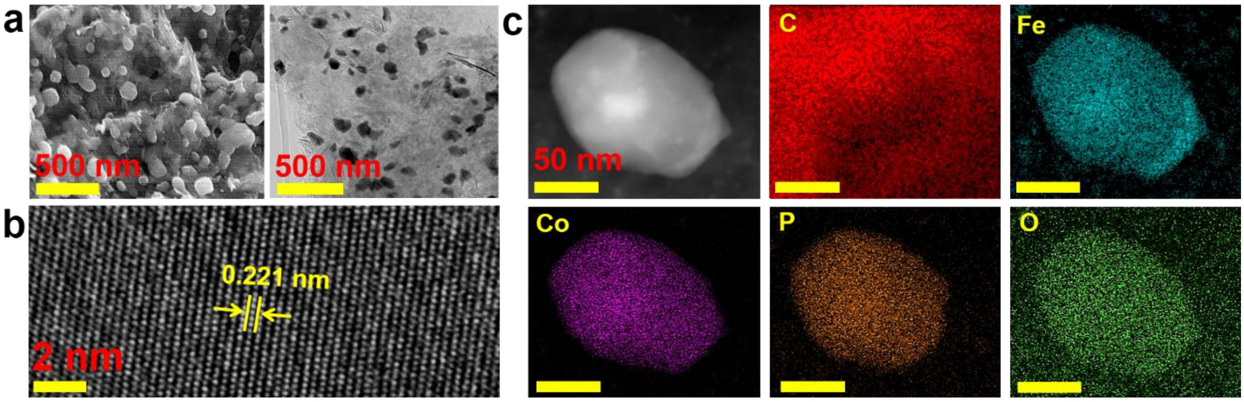

Supplementary Figure 27 | Structural and compositional characterizations of $FeCo(PO_4)_2$@rGO (2). a, Scanning (left) and transmission (right) electron micrographs. SEM and TEM images show that the diameters of NPs are in the range of 70–130 nm **b,** High-resolution transmission electron microscopy (HRTEM) image of single-particle. **c,** High-angle annular dark field-scanning transmission electron microscope (HAADF–STEM) image and their corresponding individual element maps of C, Fe, Co, P, and O.

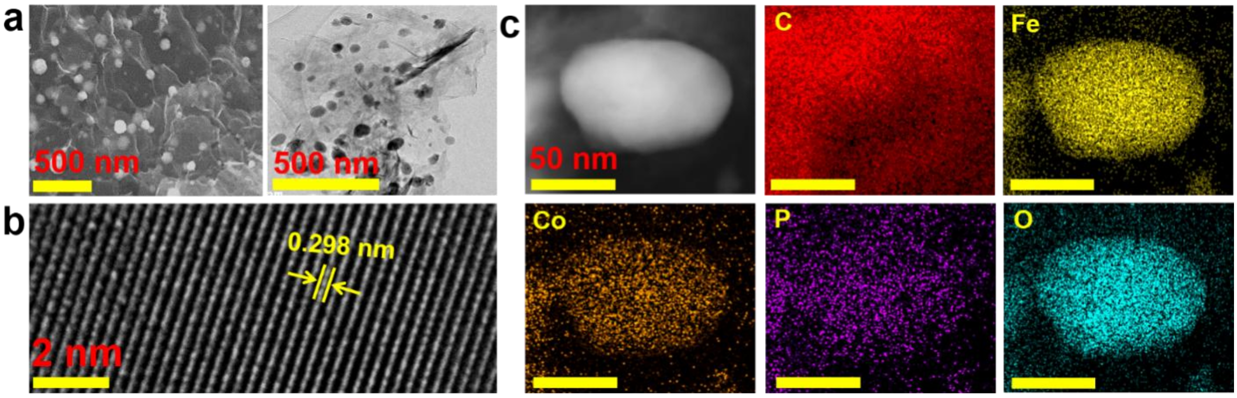

Supplementary Figure 28 | Structural and compositional characterizations of $Fe_2Co(PO_4)_3$@rGO (3). a, Scanning (left) and transmission (right) electron micrographs. SEM and TEM images show that the diameters of NPs are in the range of 60–95 nm **b,** High-resolution transmission electron microscopy (HRTEM) image of single-particle. **c,** High-angle annular dark field-scanning transmission electron microscope (HAADF–STEM) image and their corresponding individual element maps of C, Fe, Co, P, and O.

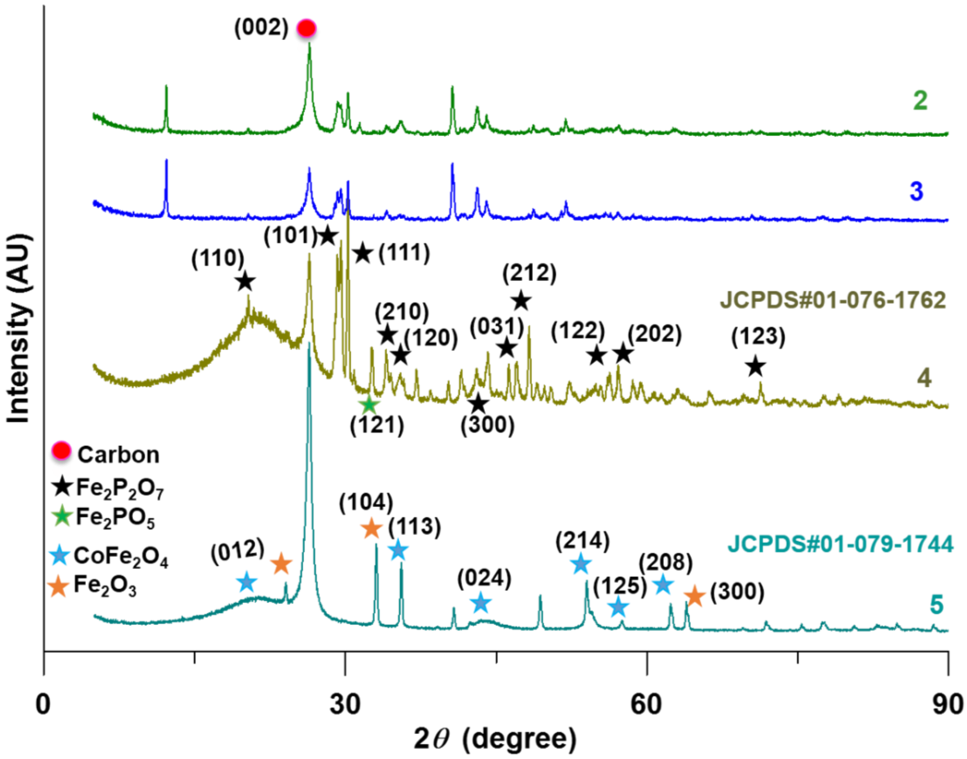

Supplementary Figure 29 | X−ray diffraction (XRD) characterization. XRD patterns for catalysts of **2**: $FeCo(PO_4)_2$@rGO, **3**: $Fe_2Co(PO_4)_3$@rGO, **4:** ($Fe_2P_2O_7$)@rGO, and **5**:($CoFe_2O_4$)(Fe_2O_3)@rGO (refer to **Supplementary Tables 4 and 5**).

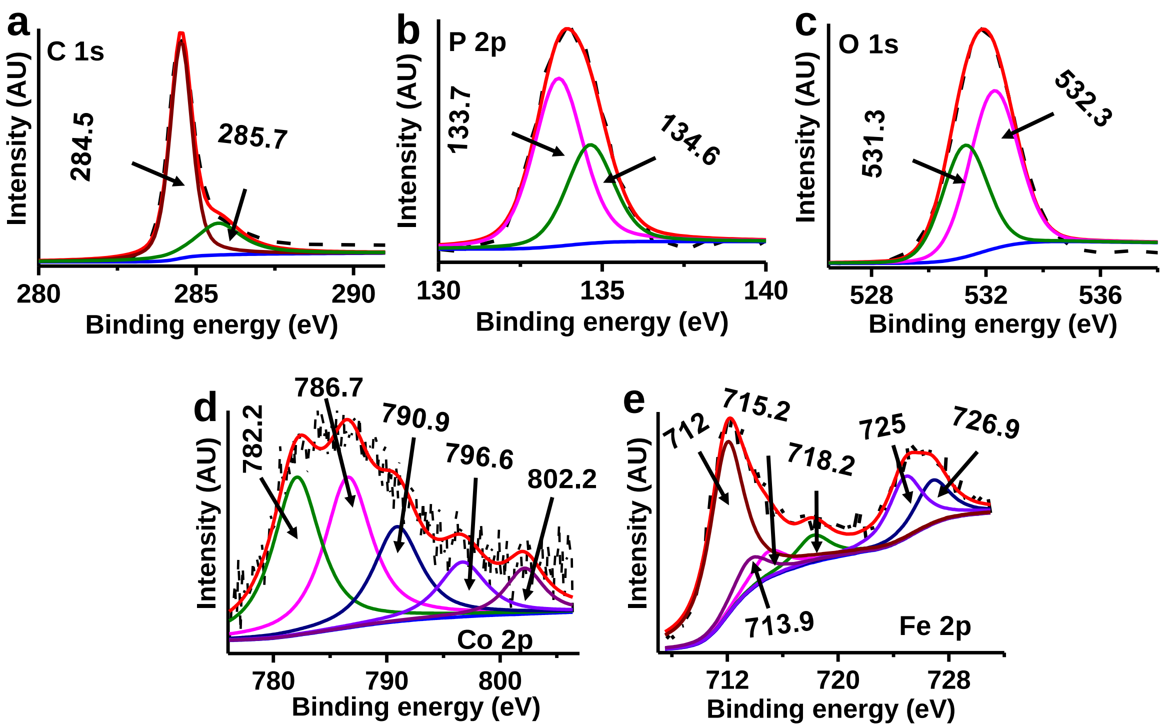

Supplementary Figure 30 | XPS core-level spectra of catalyst 2. a, C 1s; **b,** P 2p; **c,** O 1s; **d,** Co 2p; **e,** Fe 2p.

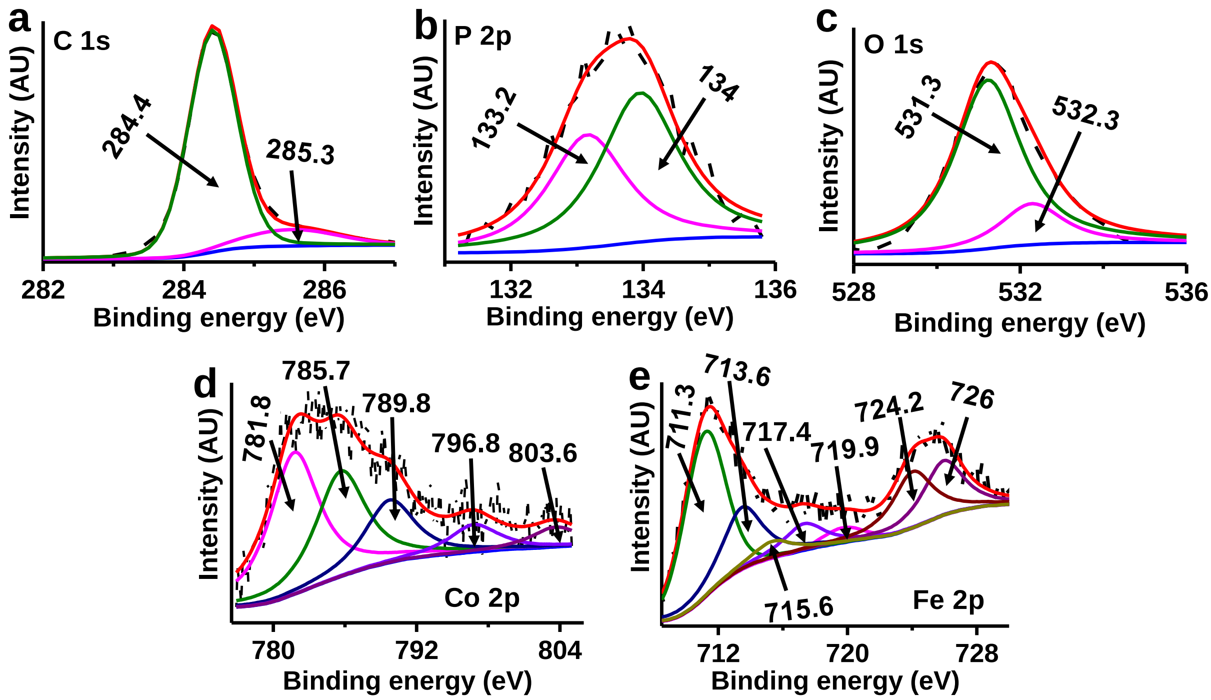

Supplementary Figure 31 | XPS core-level spectra of catalyst 3. a, C 1s; **b,** P 2p; **c,** O 1s; **d,** Co 2p; **e,** Fe 2p.

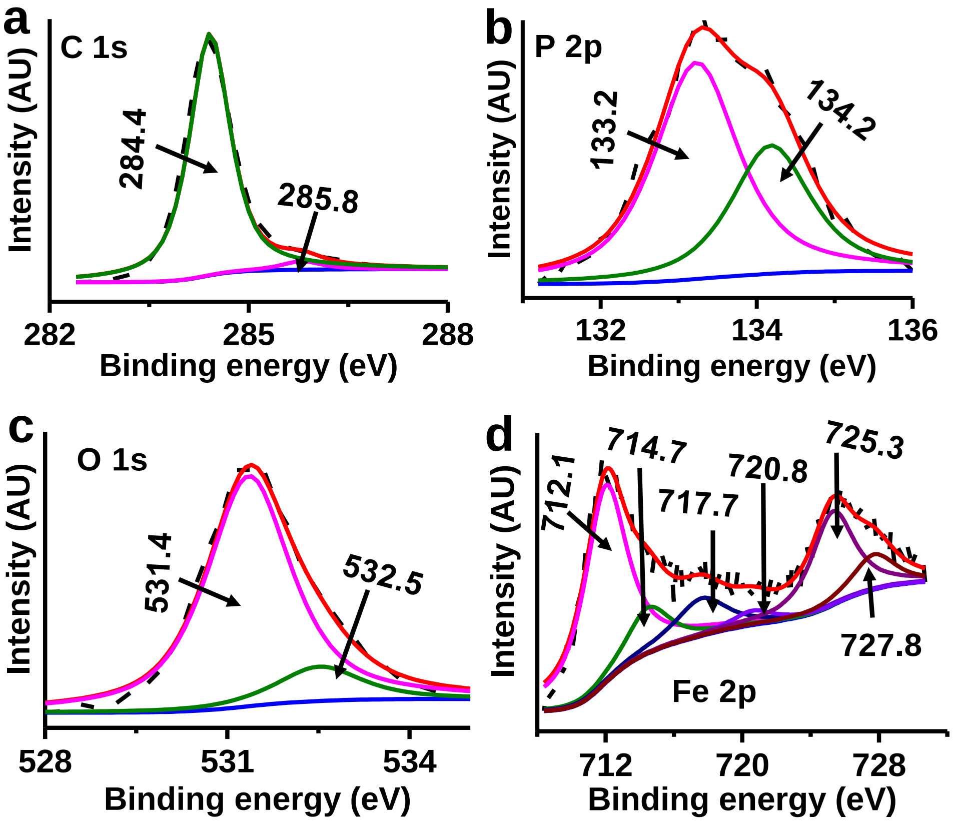

Supplementary Figure 32 | XPS core-level spectra of catalyst 4. a, C 1s; **b,** P 2p; **c,** O 1s; **d,** Fe 2p.

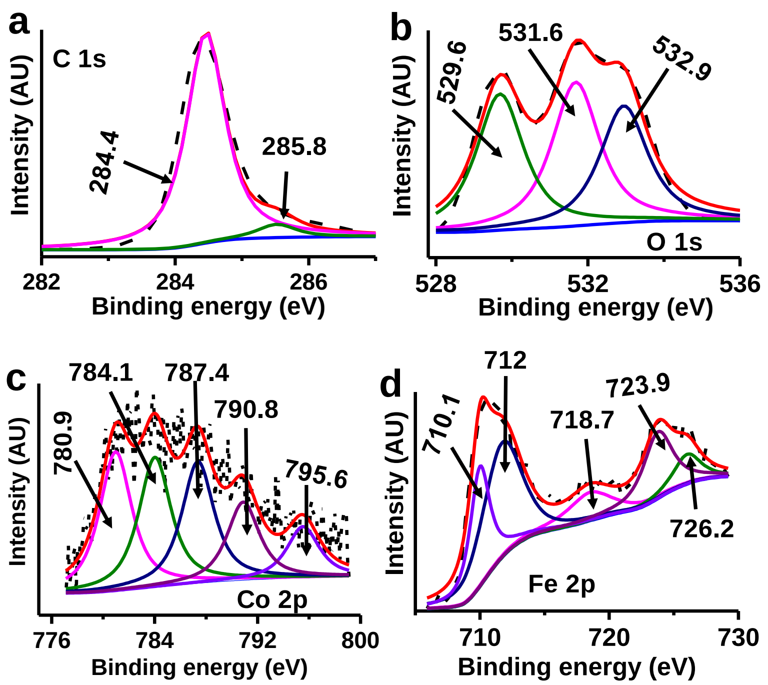

Supplementary Figure 33 | XPS core-level spectra of catalyst 5 ($Fe_xCo_yO_z$@rGO). a, C 1s; **b,** O 1s; **c,** Co 2p; **d,** Fe 2p.

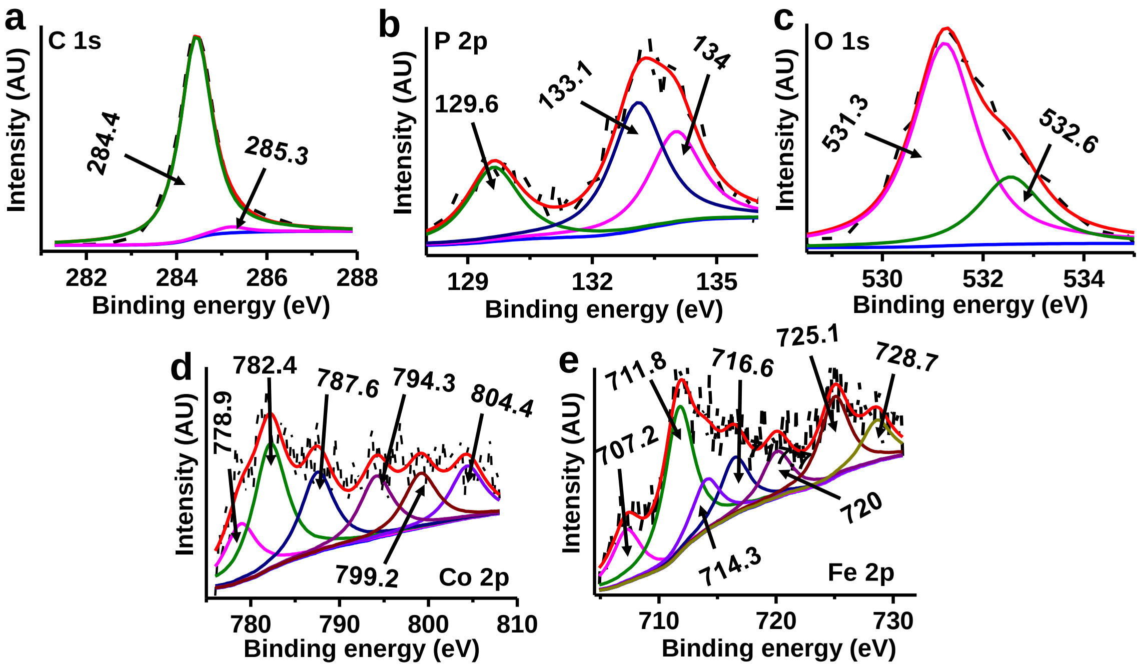

Supplementary Figure 34 | XPS core-level spectra of catalyst 6. a, C 1s; **b,** P 2p; **c,** O 1s; **d,** Co 2p; **e,** Fe 2p.

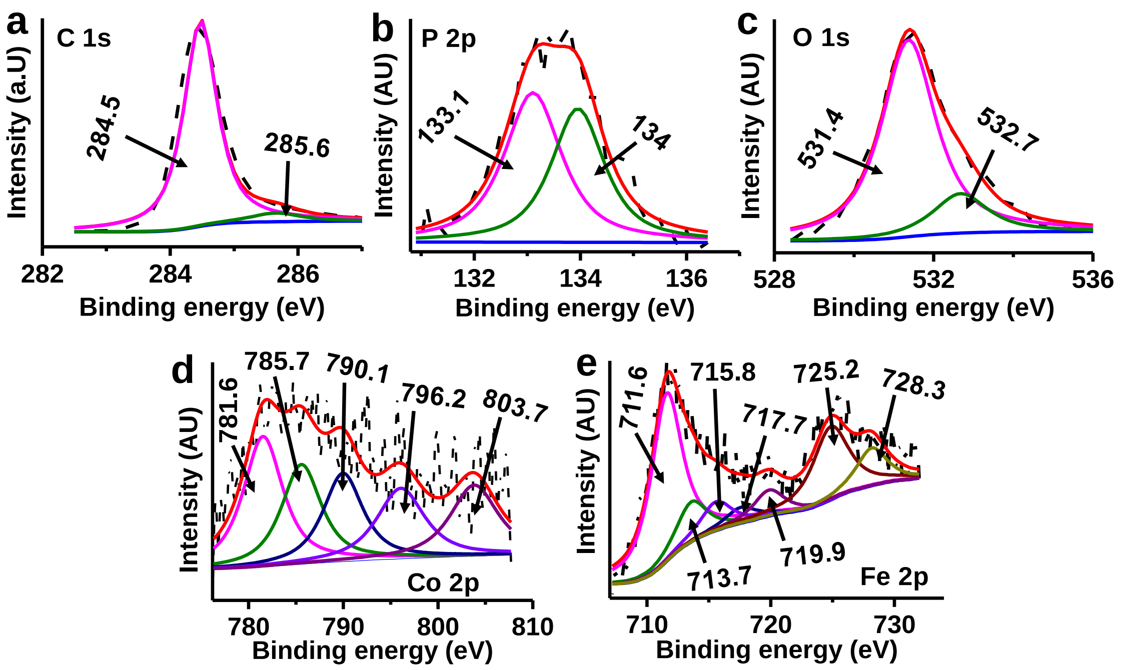

Supplementary Figure 35 | XPS core-level spectra of catalyst 7. a, C 1s; **b,** P 2p; **c,** O 1s; **d,** Co 2p; **e,** Fe 2p.

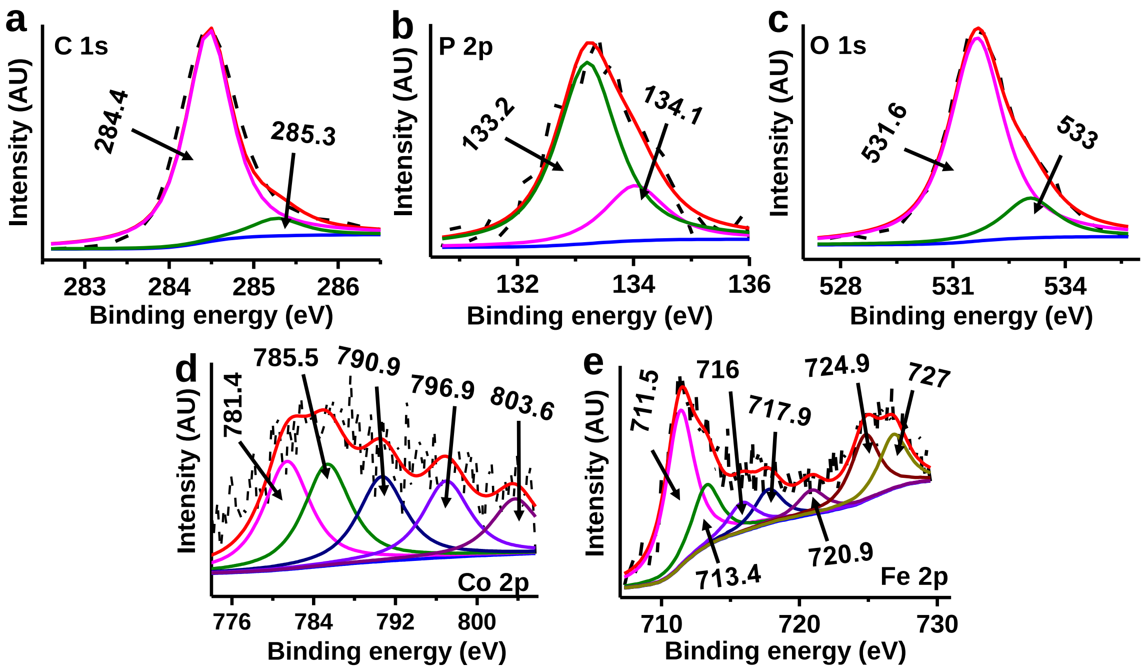

Supplementary Figure 36 | XPS core-level spectra of catalyst 8. a, C 1s; **b,** P 2p; **c,** O 1s; **d,** Co 2p; **e,** Fe 2p.

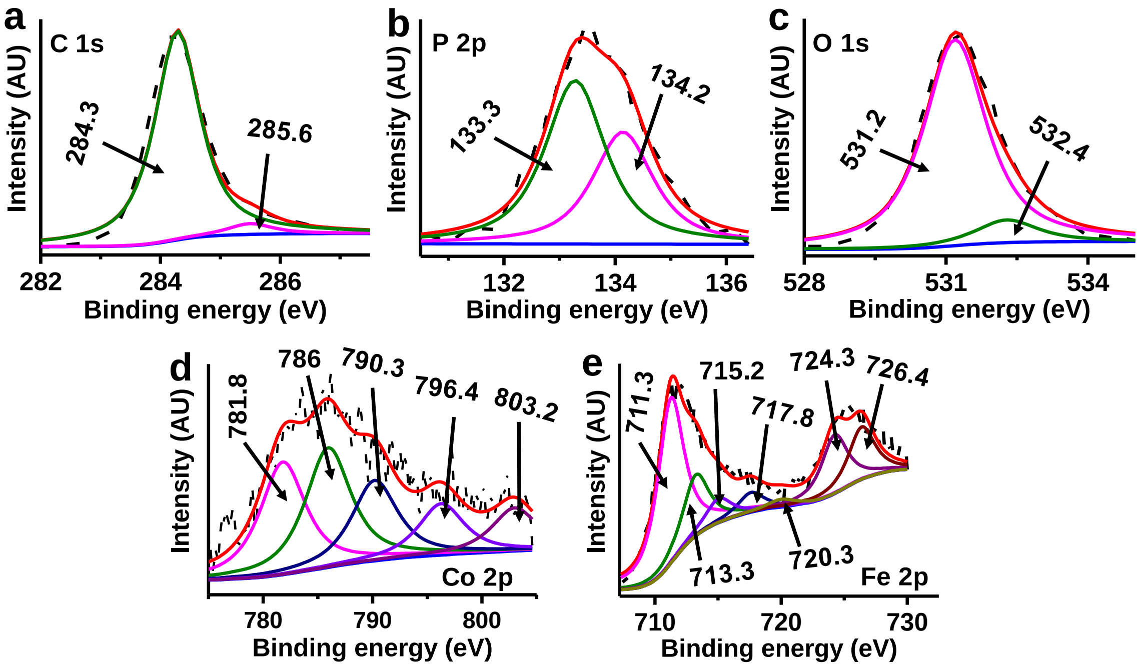

Supplementary Figure 37| XPS core-level spectra of catalyst 9. a, C 1s; **b,** P 2p; **c,** O 1s; **d,** Co 2p; **e,** Fe 2p.

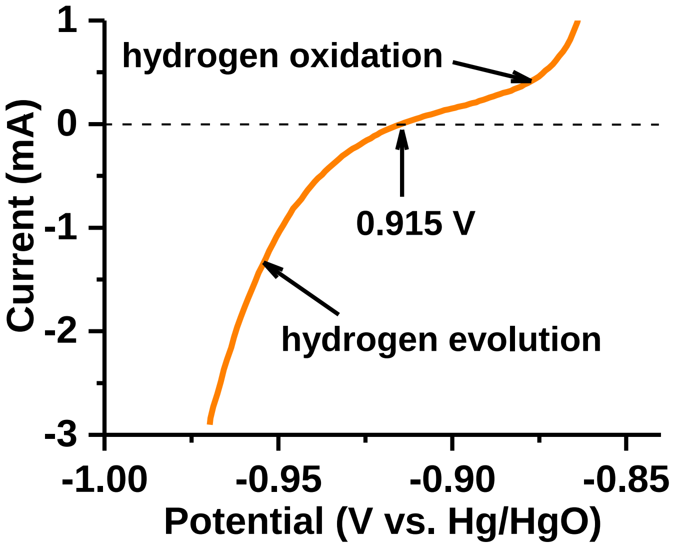

Supplementary Figure 38 | Calibration of reference electrode. Calibration curve for Hg/HgO electrode in 1M KOH.

Supplementary Tables

Supplementary Table 1 | DFT-predicted Gibbs free energies for OER steps (intermediate states) and overpotential (η^{theory}) at Fe and Co sites of various composites (see the scheme in computational method).

Supplementary Table 2 | Surface energies (γ) of $Fe_3Co(PO_4)_4$. $\gamma = (E_{surf} - n\times E_{bulk})/2A$ where E_{surf} is total energy of each surface including n formula units, E_{bulk} is total energy per formula unit of $Fe_3Co(PO_4)_4$, and A is surface area. Miller index is based on the $Fe_3Co(PO_4)_4$ bulk unit cell [5].

Surface	γ ($J\cdot m^{-2}$)
(010)	0.36
(100)	0.63
(101)	0.64
(111)	0.84
(001)	0.93
(110)	1.04

Supplementary Table 3 | Curve-fitting analysis for Fe K-edge EXAFS of $Fe_3Co(PO_4)_4$@rGO before and after OER test. Fe K-edge EXAFS curve fitting parameters (R: Bond distance, CN: Coordination number, σ^2: Debye-Waller factor, ΔE_0: energy shift).

**Before OER test**				
R-factor	0.0082			
Path	R(Å)	CN (atoms)	σ^2 (Å^2)	ΔE_0
Fe-O	2.02 ± 0.03	4.1	0.010 ± 0.002	4.98 ± 2.52
Fe-O	2.23 ± 0.03	1.9	0.010 ± 0.002	4.98 ± 2.52
Fe-P	2.88 ± 0.06	1.6	0.010 ± 0.004	4.98 ± 2.52
Fe-P	3.11 ± 0.18	1	0.010 ± 0.004	4.98 ± 2.52
Fe-P	3.31 ± 0.07	2.4	0.010 ± 0.004	4.98 ± 2.52

**After OER test**				
R-factor	0.0082			
Path	R(Å)	CN (atoms)	σ^2 (Å^2)	ΔE_0
Fe-O	2.00 ± 0.02	4.8	0.012 ± 0.001	4.08 ± 2.65
Fe-O	2.21 ± 0.02	1.2	0.012 ± 0.001	4.08 ± 2.65
Fe-P	2.75 ± 0.04	1.3	0.006 ± 0.003	4.08 ± 2.65
Fe-P	2.94 ± 0.11	1	0.006 ± 0.003	4.08 ± 2.65
Fe-P	3.19 ± 0.03	2.7	0.006 ± 0.003	4.08 ± 2.65

Supplementary Table 4 | Curve-fitting analysis for Co K-edge EXAFS of $Fe_3Co(PO_4)_4$@rGO before and after OER test. Co K-edge EXAFS curve fitting parameters (R: Bond distance, CN: Coordination number, σ^2: Debye-Waller factor, ΔE_0: energy shift).

**Before OER test**				
R-factor	0.0025			
Path	R(Å)	CN (atoms)	σ^2 (Å^2)	ΔE_0
Co-O	1.98 ± 0.01	2	0.0058 ± 0.0008	-2.56 ± 1.07
Co-O	2.39 ± 0.01	4	0.0058 ± 0.0008	-2.56 ± 1.07
Co-P	2.81 ± 0.01	5	0.0039 ± 0.0005	-2.56 ± 1.07

**After OER test**				
R-factor	0.0041			
Path	R(Å)	CN (atoms)	σ^2 (Å^2)	ΔE_0
Co-O	1.94 ± 0.01	2	0.006 ± 0.001	-7.34 ± 1.37
Co-O	2.35 ± 0.01	4	0.006 ± 0.001	-7.34 ± 1.37
Co-P	2.79 ± 0.01	5	0.007 ± 0.001	-7.34 ± 1.37

Supplementary Table 5 | ICP-AES Chemical composition analysis. ICP-AES analysis gives the atomic percentage of bulk sample. The analysis of C and O is not possible through ICP-AES, while the content of P tends to be underestimated compared with metals. Thus, only contents of Co and Fe are investigated here. Among four different samples, two mid-value data are reported, and the Fe/Co compositions of samples **1-3** are roughly determined.

Catalysts	Co (g/kg)	Fe (g/kg)	Co:Fe [at. ratio]	composition
**1**	81	233	1: 3.0	$CoFe_3$
	66.5	197	1: 3.1	$CoFe_3$
**2**	129	130	1: 1.1	CoFe
	114	136	1: 1.3	~ Co_3Fe_4
**3**	91	128	1: 1.5	Co_2Fe_3
	83	144	1: 1.8	~ $CoFe_2$

Supplementary Table 6 | XPS Chemical composition analysis. XPS analysis provides the atomic percentage near sample surface. As compared with ICP-AES bulk sample analysis, the XPS surface analysis increases the $Fe(PO_4)$ content, while the atomic content of P (or PO_4) is almost the sum of Co and Fe atomic contents, indicating the charges of Co and Fe are +3. The content of O is slightly larger than 4 times of the content of P due to environmental oxygen. An extra content of $Fe(PO_4)$, as noted from XPS over ICP-AES could be present on the surface more than in bulk. However, the XPS data are not so reliable for accurate composition analysis as compared with ICP-AES. The Fe/Co metals composition ratio from ICP-AES is more reliable.

**Catalysts**	**Co**	**Fe**	**P**	**O**	**C**	**Co:Fe:P:O**	**Composition**
**1**	1.07	4.02	4.86	21.31	68.73	1: 3.8: 4.5: 19.9	$CoFe_3(PO_4)_4$+$Fe(PO_4)$
**2**	1.09	2.32	3.80	20.06	70.99	1: 2.1: 3.5: 18.4	$CoFe(PO_4)_2$+$Fe(PO_4)$
**3**	0.941	2.95	4.37	18.91	72.48	1: 3.1: 4.6: 20.1	$CoFe_2(PO_4)_3$+$Fe(PO_4)$

Supplementary Table 7 | XPS Chemical composition analysis of catalysts 6-9. The atomic content of C and P obtained from XPS analysis in Catalysts **6-9**.

Catalysts	C	P
**6**	53.04	4.59
**7**	82.28	2.96
**8**	76.23	2.56
**9**	67.14	6.09

Supplementary Table 8 | Best OER activity in alkaline solution. Performance of Fe-, Co-, and phosphide-based OER elctrocatalysts reported in the recent literature.

Catalysts	Overpotential (mV) **@100** mA cm^{-2}	Refs.
**Catalysts with C**		
$Fe_3Co(PO_4)_4$@rGO/NF	237	this work
Commercial 20 % Ir/C	303	this work
CoP/NCNHP	370 @80 $mAcm^{-2}$	11
CoP NR/C	530	12
CoP@rGO-400	470	13
Mn-NG	337 @10 $mAcm^{-2}$	14
Fe_3N/Fe_4N	295	15
Fe-Co-P	270 @45 $mAcm^{-2}$	16
Ni-P	320 @35 $mAcm^{-2}$	17
NiFe-PBA	330	18
Fe_3Co_2@Ni	280	19
INONFs-45	450	20
2.5H-PHNCMs	370	21
HG-NiFe	380	22
**Catalysts without C**		
FeCoW/Au-foam†	180 @10 $mAcm^{-2}$<br>250 @100 $mAcm^{-2}$	23
Core-shell FeNiCu	180 @10 $mAcm^{-2}$<br>190 @20 $mAcm^{-2}$<br>230 @100 $mAcm^{-2}$	24
$Co_{0.93}Ni_{0.07}P_3$	360	25
CoFe-0.44	281	26
CoNi(20:1)-P-NS@NF	253	27
Co_3Ni_1P	370	28
Au/NiFe LDH	280	29
NiFeP	360	30
$Ni_{95}Ce5O_x$-Au†	350 @70 $mAcm^{-2}$	31
$Ni_3Fe_{0.5}V_{0.5}$	260	32
NiFe/Ni-P	260	33
FeCoNi-HNTAs	390	34

Supplementary Table 9 | Calculated atomic magnetic moment in μ_B. Both Fe and Co atoms in $Fe_xCo_{4-x}(PO_4)_4$ (x=1-4) have high spin states.

Material	**μ_B (Fe)**	**μ_B (Co)**
$Fe_4(PO_4)_4$ (010)	4.01	-
$Fe_3Co(PO_4)_4$ (010)	3.98	2.89
$Fe_2Co_2(PO_4)_4$ (010)	3.97	2.90
$FeCo_3(PO_4)_4$ (010)	3.93	2.73
$Co_4(PO_4)_4$ (010)	-	2.86

Supplementary Notes

Supplementary Note 1 | Calibration of reference electrode.

Reference electrode calibration was carried in a three electrode system with Pt foil as working and counter electrode and Hg/HgO (1M NaOH) as reference electrode. The calibration was performed in high purity hydrogen saturated 1M KOH electrolyte. Steady-state linear-sweep voltammetry (LSV) was run at a scan rate of 0.5 mV s^{-1} and the potential at which current crosses zero was taken as thermodynamic potential (vs. Hg/HgO) for the hydrogen electrode (**Supplementary Fig. 38**).

The potential at which current crosses zero is -0.915 V vs Hg/HgO.

Thus, E (reversible hydrogen electrode; RHE) = E(Hg/HgO) + 0.915 V.

Supplementary Note 2 | Characterization of electrocatalysts.

Scanning and transmission electron micrographs of $FeCo(PO_4)_2$@rGO and $Fe_2Co(PO_4)_3$@rGO. The SEM and TEM images show that nanoparticles (NPs) in both $FeCo(PO_4)_2$@rGO and $Fe_2Co(PO_4)_3$@rGO are uniformly distributed on the rGO surface **(Supplementary Figs. 27a & 28a)**. From the TEM images, we note that the diameters of NPs increase with increasing cobalt content in the $Fe_xCo_y(PO_4)_{x+y}$@rGO **(Supplementary Figs. 27a & 28a)**. The HR-TEM images demonstrate that both NPs are crystalline (**Supplementary Figs. 27b & 28b)**. Furthermore, the HRTEM image of $FeCo(PO_4)_2$@rGO shows the d-spacing of ~0.221 nm (**Supplementary Figs. 27b**), while in $Fe_2Co(PO_4)_3$@rGO the d-spacing is ~0.298 nm **(Supplementary Figs. 28b)**. The elements distribution of both NPs are examined by high-angle annular-dark-field scanning-TEM energy-dispersive spectroscopy (HAADF-STEM-EDS), **(Supplementary Figs. 27c & 28c)**.

X-ray diffraction (XRD). The powder X-ray diffraction (XRD) patterns of as-synthesized **2** ($FeCo(PO_4)_2$@rGO), **3** ($Fe_2Co(PO_4)_3$@rGO), **4** (($Fe_2P_2O_7$)@rGO), and **5** ($CoFe_2O_4$)(Fe_2O_3)@rGO) are characterized, which confirms the formation of crystalline phase (**Supplementary Fig. 29**). The XRD peaks (except one peak at 32.8^0) of **4** match with the standard PDF card of $Fe_2P_2O_7$@rGO (JCPDS 01-076-1762). The peak at 32.8^0 matches with the PDF card of Fe_2PO_5 (JCPDS 00-036-0084). The XRD pattern of **5** shows a mixture of $CoFe_2O_4$ and Fe_2O_3, in good agreement with the standard data (JCPDS 01-079-1744 for $CoFe_2O_4$ and 01-079-1744 for Fe_2O_3). Consequently, XRD patterns of **2-5** indicate the formation of desired products with the highest degree of crystallinity.

Core level XPS spectra of 1 (Fe_3Co $(PO_4)_4$@rGO). The core level XPS spectrum of C 1s shows the main strong and sharp peak at 284.6 eV corresponds to graphitic carbon, while the peak located at 285.8 eV is assigned to C-O/C-P (**Supplementary Fig. 20a**)[35]. The spectrum of Co 2p displays the core-level XPS peaks at 782.3 eV (2p3/2) and 797.4 eV (2p1/2) with satellite peaks at 786.6, 790.7, and 803 eV corresponding to the cationic state of Co species (**Supplementary Fig. 20b**)[12]. The Fe 2p spectrum exhibits peaks of two different spin-orbits. The peaks located at binding energies of 713.2 and 725.3 eV with shakeup satellites (718.8 and 728 eV) are attributed to $2p_{3/2}$ and the $2p_{1/2}$ of Fe^{3+}, while the peak located at binding energy of 711.6 eV with satellite peak of 715.3 eV correspond to Fe^{2+} state (**Supplementary Fig. 20c**)[36, 37, 38].

Core level XPS spectra of 2 ($FeCo(PO_4)_2$@rGO). In the core level XPS spectrum of C 1s, the peak centered at 284.5 eV corresponds to the graphitic carbon, while the peak located at 285.7 eV is assigned to C-O/C-P (**Supplementary Fig. 30a**)[35]. The core level XPS spectrum of P 2p shows typical peaks of phosphate species at binding energies of 133.7 and 134.6 eV (**Supplementary Fig. 30b**)[39]. The peaks of O 1s at binding energies of 531.3 and 532.3 eV correspond to the core level of O in phosphate group (**Supplementary Fig. 30c**)[39]. The XPS spectrum of Co 2p shows two core-level peaks at 782.2 eV (2p3/2) and 796.6 eV (2p1/2) with satellite peaks at 786.7, 790.9, and 802.2 eV corresponding to the cationic state of Co species (**Supplementary Fig. 30d**)[12]. The XPS spectrum of Fe 2p shows peaks of two different spin-orbits. The peaks at binding energy of 713.9 and 725 eV with shakeup satellites (718.2 and 726.9 eV) are assigned to $2p_{3/2}$ and the $2p_{1/2}$ of Fe^{3+}, while the peak located at binding energy of 712 eV with satellite peak of 715.2 eV correspond to Fe^{2+} state (**Supplementary Fig. 30e**)[37, 38].

Core level XPS spectra of 3 ($Fe_2Co(PO_4)_3$@rGO). The core level XPS spectrum of C 1s shows the peak of graphitic carbon at binding energy of 284.4 eV and C-O/C-P at binding energy of 285.3 eV (**Supplementary Fig. 31a**)[35]. The high resolution XPS spectrum of P 2p shows the phosphate peaks at binding energies of 133.2 and 134 eV (**Supplementary Fig. 31b**)[39]. Similarly, the XPS spectrum of O 1s in phosphate group shows the peaks at binding energies of 531.3 and 532.3 eV (**Supplementary Fig. 31c**)[39]. The spectrum of Co 2p shows the peaks of $2p_{3/2}$ and $2p_{1/2}$ at binding energies of 781.8 eV and 796.8 eV with shakeup satellite peaks at 785.7, 789.8, and 803.6 eV (**Supplementary Fig. 31d**)[12]. The Fe 2p XPS spectrum exhibits the peaks of Fe^{+3} at binding energies of 713.6 and 724.2 eV with satellites peaks of (719.9 and 726 eV) and peak of Fe^{2+} at binding energy of 711.3 eV with satellites peak of 715.6 and 717.4 eV (**Supplementary Fig. 31e**)[37, 38].

Core level XPS spectra of 4 ($Fe_2P_2O_7$@rGO). The XPS spectrum of C 1s in FeP_2O_7 shows the peak of graphitic carbon at binding energy of 284.4 eV and C-O/C-P at binding energy of 285.8 eV (**Supplementary Fig. 32a**)[35]. The peaks of phosphate in FeP_2O_7 is located at binding energies of 133.2 and 134.2 eV (**Supplementary Fig. 32b**)[39]. The P-O in phosphate group is located at binding energies of 531.4 and 532.5 eV (**Supplementary Fig. 32c**)[39]. The Fe 2p in FeP_2O_7 exhibits $2p_{3/2}$ and the $2p_{1/2}$ of Fe^{3+} at binding energies of 714.7 and 725.3 eV with shakeup satellites (717.7 and 727.8 eV) and Fe^{2+} at binding energies of 712.1 and 720.8 eV (**Supplementary Fig. 32d**)[37, 38, 40].

Core level XPS spectra of 5 (($CoFe_2O_4$)(Fe_2O_3)@rGO). The XPS spectrum of C 1s in ($CoFe_2$)O_4-GO shows the peak of graphitic carbon and C-O at binding energies of 284.4 and 285.8, respectively (**Supplementary Fig. 33a**)[35]. The high resolution XPS spectrum of oxygen shows three peaks at binding energies of 529.6, 531.6, and 532.9 eV, which can be assigned to metal-oxygen bond, metal- hydroxides, and adsorbed oxygen species (**Supplementary Fig. 33b**)[41, 42]. The XPS spectrum of Co 2p displays core-level peaks at binding energies of 780.9 eV (2p3/2) and 795.6 eV (2p1/2) with satellite peaks at 784.1, 787.5, and 790.8, eV corresponding to the Co^{2+} species in ($CoFe_2$)O_4 (**Supplementary Fig. 33c**)[43, 44]. The high resolution XPS spectrum of Fe 2p in ($CoFe_2$)O_4 exhibits peaks of two different spin-orbits. The peaks at binding energies of 710.1 and 723.9 eV corresponding to Fe_3O_4, while the peak located at binding energy of 712 eV with satellite peaks of 718.7 and 726.2 eV corresponds to Fe^{3+} state (**Supplementary Fig. 33d**)[37, 38, 40].

Core level XPS spectra of Catalysts 6-9. The Supplementary Figs. 34-37 show the core level XPS spectra of C 1s, P 2p, O 1s, Co 2p and Fe 2p of catalysts **6-9**. In all these catalysts **6-9**, the

C Is have the peaks of graphitic carbon (284.3-284.5 eV) and C-O/C-P (285.3 or 285.6 eV) (**Supplementary Figs. 34a-37a**)[35]. Similarly, the XPS spectra of O 1s in catalysts **6-9** show the peaks at binding energies which can be assigned to phosphate group (**Supplementary Figs. 34c-37c)**[39]. The core-level XPS spectra of P 2p, Co 2p and Fe 2p of catalysts **7-9** have almost similar binding energy to that of catalyst **1** in which the XPS spectra of P 2p show the typical peaks of phosphate species at binding energies of 133.1-133.3 eV and 134-34.2 eV (**Supplementary Fig. 35b-37b**)[39]. The binding energies of Co and Fe spectra (**Supplementary Figs. 35d, e – 37d, e)** show that Co and Fe in catalysts **7-9** have similar cationic states to that of catalyst **1**. However, in catalyst **6** we note that the core level XPS spectra of P 2p, Co 2p and Fe 2p have some different peaks compared to catalyst **1**. For example, the core level XPS spectrum of P 2p in catalyst **6** shows two different states of peaks (**Supplementary Fig. 34b**), one can be assigned to phosphide (unresolved doublet centered at 129.6 eV)[45] and the other to phosphate (resolved doublet centered at 133.1 eV and 134 eV)[39]. The high-resolution XPS spectrum of Co 2p in catalyst **6** shows two pairs of peaks (**Supplementary Fig. 34d**). The peaks located at binding energies of 778.9 and 794.3 eV are assigned to metallic Co in CoP [46], while the peaks at binding energies of 782.4 and 799.2 eV with shakeup satellites (787.6 and 804.4 eV) correspond to cationic cobalt in metal phosphate[12]. Similarly, the core level XPS of Fe 2p in catalyst **6** shows zero valence state peaks at binding energies of 707.2 and 720 eV [46] and cationic state peaks at binding energies of 711.8, 714.3 and 725.1 eV with shakeup satellite peaks at 716.6 and 728.7 eV, which are attributed to metal phosphide and metal phosphate, respectively (**Supplementary Fig. 34e**).

Supplementary References

1. Dronskowski, R. & Bloechl, P. E. Crystal orbital hamilton populations (COHP): energy-resolved visualization of chemical bonding in solids based on density-functional calculations. *J. Phys. Chem.* **97**, 8617-8624 (1993).
2. Deringer, V. L., Tchougréeff, A. L. & Dronskowski, R. Crystal orbital hamilton population (COHP) analysis as projected from plane-wave basis sets. *J. Phys. Chem. A* **115**, 5461-5466 (2011).
3. Maintz, S., Deringer, V. L., Tchougréeff, A. L. & Dronskowski, R. Analytic projection from plane-wave and PAW wavefunctions and application to chemical-bonding analysis in solids. *J. Comput. Chem.* **34**, 2557-2567 (2013).
4. Maintz, S., Deringer, V. L., Tchougréeff, A. L. & Dronskowski, R. LOBSTER: A tool to extract chemical bonding from plane-wave based DFT. *J. Comput. Chem.* **37**, 1030-1035 (2016).
5. Jain, A. et al. Commentary: the materials project: A materials genome approach to accelerating materials innovation. *APL Mater.* **1**, 011002 (2013).
6. Bunău, O. & Joly, Y. Self-consistent aspects of x-ray absorption calculations. *J. Phys. Condens. Matter* **21**, 345501 (2009).
7. Bourke, J. D., Chantler, C. T. & Joly, Y. FDMX: extended X-ray absorption fine structure calculations using the finite difference method. *J. Synchrotron Radiat.* **23**, 551-559 (2016).
8. Bajdich, M., García-Mota, M., Vojvodic, A., Nørskov, J. K. & Bell, A. T. Theoretical investigation of the activity of cobalt oxides for the electrochemical oxidation of water. *J. Am. Chem. Soc.* **135**, 13521-13530 (2013).
9. Zhang, Y., Savara, A. & Mullins, D. R. Ambient-pressure XPS studies of reactions of alcohols on SrTiO3(100). *J. Phys. Chem. C* **121**, 23436-23445 (2017).
10. Kumar, R. A., Babu, K. S., Dasgupta, A. & Ramaseshan, R. Enhancing the dual magnetic and optical properties of co-doped cerium oxide nanostructures. *RSC Adv.* **5**, 103465-103473 (2015).
11. Pan, Y. et al. Core–Shell ZIF-8@ZIF-67-Derived CoP nanoparticle-embedded N-doped carbon nanotube hollow polyhedron for efficient overall water splitting. *J. Am. Chem. Soc.* **140**, 2610-2618 (2018).
12. Chang, J. et al. Surface oxidized cobalt-phosphide nanorods as an advanced oxygen evolution catalyst in alkaline solution. *ACS Catal.* **5**, 6874-6878 (2015).
13. Jiao, L., Zhou, Y.-X. & Jiang, H.-L. Metal–organic framework-based CoP/reduced graphene oxide: high-performance bifunctional electrocatalyst for overall water splitting. *Chem. Sci.* **7**, 1690-1695 (2016).
14. Guan, J. et al. Water oxidation on a mononuclear manganese heterogeneous catalyst. *Nat. Catal.* **1**, 870-877 (2018).
15. Yu, F. et al. Three-dimensional nanoporous iron nitride film as an efficient electrocatalyst for water oxidation. *ACS Catal.* **7**, 2052-2057 (2017).

16. Liu, K. et al. High-performance transition metal phosphide alloy catalyst for oxygen evolution reaction. *ACS Nano* **12**, 158-167 (2018).

17. Yu, X.-Y., Feng, Y., Guan, B., Lou, X. W. & Paik, U. Carbon coated porous nickel phosphides nanoplates for highly efficient oxygen evolution reaction. *Energy Environ. Sci.* **9**, 1246-1250 (2016).

18. Su, X. et al. Operando spectroscopic identification of active sites in NiFe prussian blue analogues as electrocatalysts: activation of oxygen atoms for oxygen evolution reaction. *J. Am. Chem. Soc.* **140**, 11286-11292 (2018).

19. Shen, J.-Q. et al. Modular and stepwise synthesis of a hybrid metal–organic framework for efficient electrocatalytic oxygen evolution. *J. Am. Chem. Soc.* **139**, 1778-1781 (2017).

20. Fan, X. et al. Defect-enriched iron fluoride-oxide nanoporous thin films bifunctional catalyst for water splitting. *Nat. Commun.* **9**, 1809 (2018).

21. Li, H. et al. Amorphous nickel-cobalt complexes hybridized with 1T-phase molybdenum disulfide via hydrazine-induced phase transformation for water splitting. *Nat. Commun.* **8**, 15377 (2017).

22. Wang, J. et al. In situ formation of molecular Ni-Fe active sites on heteroatom-doped graphene as a heterogeneous electrocatalyst toward oxygen evolution. *Sci. Adv.* **4**, eaap7970 (2018).

23. Zhang, B. et al. Homogeneously dispersed multimetal oxygen-evolving catalysts. *Science* **352**, 333 (2016).

24. Zhang, P. et al. Dendritic core-shell nickel-iron-copper metal/metal oxide electrode for efficient electrocatalytic water oxidation. *Nat. Commun.* **9**, 381 (2018).

25. Fu, Q. et al. Skutterudite-type ternary $Co_{1-x}Ni_xP_3$ nanoneedle array electrocatalysts for enhanced hydrogen and oxygen evolution. *ACS Energy Lett.* **3**, 1744-1752 (2018).

26. Hung, S.-F. et al. unraveling geometrical site confinement in highly efficient iron-doped electrocatalysts toward oxygen evolution reaction. *Adv. Energy Mater.* **8**, 1701686 (2018).

27. Xiao, X. et al. A general approach to cobalt-based homobimetallic phosphide ultrathin nanosheets for highly efficient oxygen evolution in alkaline media. *Energy Environ. Sci.* **10**, 893-899 (2017).

28. Fu, S. et al. Highly ordered mesoporous bimetallic phosphides as efficient oxygen evolution electrocatalysts. *ACS Energy Lett.* **1**, 792-796 (2016).

29. Zhang, J. et al. Single-atom Au/NiFe layered double hydroxide electrocatalyst: probing the origin of activity for oxygen evolution reaction. *J. Am. Chem. Soc.* **140**, 3876-3879 (2018).

30. Hu, F. et al. Amorphous metallic NiFeP: A conductive bulk material achieving high activity for oxygen evolution reaction in both alkaline and acidic Media. *Adv. Mater.* **29**, 1606570 (2017).

31. Ng, J. et al. Gold-supported cerium-doped NiO_x catalysts for water oxidation. *Nat. Energy* **1**, 16053 (2016).

32. Jiang, J. et al. Atomic-level insight into super-efficient electrocatalytic oxygen evolution on iron and vanadium co-doped nickel (oxy)hydroxide. *Nat. Commun.* **9**, 2885 (2018).

33. Sahasrabudhe, A., Dixit, H., Majee, R. & Bhattacharyya, S. Value added transformation of ubiquitous substrates into highly efficient and flexible electrodes for water splitting. *Nat. Commun.* **9**, 2014 (2018).

34. Li, H. et al. Systematic design of superaerophobic nanotube-array electrode comprised of transition-metal sulfides for overall water splitting. *Nat. Commun.* **9**, 2452 (2018).

35. Ma, J. et al. Polyaniline derived N-doped carbon-coated cobalt phosphide nanoparticles Deposited on N-Doped Graphene as an Efficient Electrocatalyst for Hydrogen Evolution Reaction. *Small* **14**, 1702895 (2017).

36. Qi, H., Qian, C. & Liu, J. Synthesis of Uniform Double-Walled Carbon Nanotubes Using Iron Disilicide as Catalyst. *Nano Lett.* **7**, 2417-2421 (2007).

37. Liu, M. et al. Fe_2P/reduced graphene oxide/Fe_2P sandwich-structured nanowall arrays: a high-performance non-noble-metal electrocatalyst for hydrogen evolution. *J. Mater. Chem.A* **5**, 8608-8615 (2017).

38. Zhang, Y., Wang, W., Li, P., Fu, Y. & Ma, X. A simple solvothermal route to synthesize graphene-modified $LiFePO_4$ cathode for high power lithium ion batteries. *J. Power Sources* **210**, 47-53 (2012).

39. Huang, J. et al. A new member of electrocatalysts based on nickel metaphosphate nanocrystals for efficient water oxidation. *Adv. Mater.* **30**, 1705045 (2017).

40. Zeng, X. et al. Single-Atom to Single-Atom Grafting of Pt_1 onto Fe-N_4 Center: Pt_1@Fe-N-C Multifunctional electrocatalyst with significantly enhanced properties. *Adv. Energy Mater.* **8**, 1701345 (2017).

41. Nardi, K. L., Yang, N., Dickens, C. F., Strickler, A. L. & Bent, S. F. Creating highly active atomic layer deposited NiO eectrocatalysts for the oxygen evolution reaction. *Adv. Energy Mater.* **5**, 1500412 (2015).

42. Gao, W. et al. Comprehensive understanding of the spatial configurations of CeO_2 in NiO for the electrocatalytic oxygen evolution reaction: embedded or surface-loaded. *Adv. Funct. Mater.* **28**, 1706056 (2018).

43. Ye, C. et al. One-step CVD synthesis of carbon framework wrapped Co_2P as a flexible electrocatalyst for efficient hydrogen evolution. *J. Mater. Chem.A* **5**, 7791-7795 (2017).

44. Chen, P. et al. Enhanced catalytic activity in nitrogen-anion modified metallic cobalt disulfide porous nanowire arrays for hydrogen evolution. *ACS Catal.* **7**, 7405-7411 (2017).

45. Li, D., Baydoun, H., Kulikowski, B. & Brock, S. L. Boosting the catalytic performance of iron phosphide nanorods for the oxygen evolution reaction by incorporation of manganese. *Chem. Mater.* **29**, 3048-3054 (2017).

46. Sultan, S. et al. Highly efficient oxygenreduction reaction activity of graphitic tube encapsulating nitrided Co_xFe_y Alloy. *Adv. Energy Mater.* **8**, 1801002 (2018).
